# Differential bioreactivity of neutral, cationic and anionic polystyrene nanoparticles with cells from the human alveolar compartment: robust response of alveolar type 1 epithelial cells

**DOI:** 10.1186/s12989-015-0091-7

**Published:** 2015-07-02

**Authors:** Pakatip Ruenraroengsak, Teresa D. Tetley

**Affiliations:** Lung Cell Biology, Section of Airways Disease, National Heart & Lung Institute, Imperial College London, Dovehouse Street, London, SW3 6LY UK

**Keywords:** Polystyrene nanoparticles, Amine-modified nanoparticles, Mitochondria, Oxidative stress, Surface modification, Human alveolar epithelial cells, Alveolar macrophages

## Abstract

**Background:**

Engineered nanoparticles (NP) are being developed for inhaled drug delivery. This route is non-invasive and the major target; alveolar epithelium provides a large surface area for drug administration and absorption, without first pass metabolism. Understanding the interaction between NPs and target cells is crucial for safe and effective NP-based drug delivery. We explored the differential effect of neutral, cationic and anionic polystyrene latex NPs on the target cells of the human alveolus, using primary human alveolar macrophages (MAC) and primary human alveolar type 2 (AT2) epithelial cells and a unique human alveolar epithelial type I-like cell (TT1). We hypothesized that the bioreactivity of the NPs would relate to their surface chemistry, charge and size as well as the functional role of their interacting cells *in vivo*.

**Methods:**

Amine- (ANP) and carboxyl- surface modified (CNP) and unmodified (UNP) polystyrene NPs, 50 and 100 nm in diameter, were studied. Cells were exposed to 1–100 μg/ml (1.25-125 μg/cm^2^; 0 μg/ml control) NP for 4 and 24 h at 37 °C with or without the antioxidant, N-acetyl cysteine (NAC). Cells were assessed for cell viability, reactive oxygen species (ROS), oxidised glutathione (GSSG/GSH ratio), mitochondrial integrity, cell morphology and particle uptake (using electron microscopy and laser scanning confocal microscopy).

**Results:**

ANP-induced cell death occurred in all cell types, inducing increased oxidative stress, mitochondrial disruption and release of cytochrome C, indicating apoptotic cell death. UNP and CNP exhibited little cytotoxicity or mitochondrial damage, although they induced ROS in AT2 and MACs. Addition of NAC reduced epithelial cell ROS, but not MAC ROS, for up to 4 h. TT1 and MAC cells internalised all NP formats, whereas only a small fraction of AT2 cells internalized ANP (not UNP or CNP). TT1 cells were the most resistant to the effects of UNP and CNP.

**Conclusion:**

ANP induced marked oxidative damage and cell death *via* apoptosis in all cell types, while UNP and CNP exhibited low cytotoxicity *via* oxidative stress. MAC and TT1 cell models show strong particle-internalization compared to the AT2 cell model, reflecting their cell function *in vivo*. The 50 nm NPs induced a higher bioreactivity in epithelial cells, whereas the 100 nm NPs show a stronger effect on phagocytic cells.

**Electronic supplementary material:**

The online version of this article (doi:10.1186/s12989-015-0091-7) contains supplementary material, which is available to authorized users.

## Background

The emergence of nanotechnology and nanomedicine is of increasing interest, particularly for local and systemic treatment *via* inhaled drug delivery to the lung [1–4]; a range of NP-based agents have been developed to improve therapeutic and diagnostic efficiency, and to minimize adverse effects [5–8]. These products have been studied *in vivo* [9–11], and also in clinical trials and some have reached the clinic for the treatment of cancer, diabetes, and other lung diseases [6, 8, 12, 13] with varying degrees of success, related to a range of factors, including the unique physicochemical structure of each type of NP and its bioreactivity. Administration of drugs *via* the lung can be performed non-invasively offering several advantages: the thin alveolar epithelial-endothelial barrier provides a large surface area with extensive vascularisation for effective drug absorption, low endogenous biotransformation activity and the drug will escape first pass metabolism in the liver [2, 3, 14]. Despite the increased use of inhalation of NPs for drug delivery [3, 15], little is known of the impact of engineered NPs on the alveolar epithelial barrier [7, 16]. It is suggested that deposition of both anthropogenic and engineered nano-sized particles could cause lung inflammation *via* oxidative stress, relating to their physicochemical properties [17, 18].

The alveolar respiratory unit is composed of alveolar type I (AT1) and type II (AT2) epithelial cells and alveolar macrophages (MAC). AT1 cells share a fused basement membrane with capillary endothelium to form a thin wall at the gas-blood barrier that facilitates gas exchange. AT2 cells secrete a range of molecules involved in lung defence and homeostasis, including lung surfactant which maintains reduced surface tension to prevent alveolar collapse; AT2 cells also proliferate and differentiate into AT1 cells to replace injured AT1 cells and have recently been described as an alveolar epithelial stem cell [19]. Alveolar macrophages (MAC) are responsible for removing foreign particles and other debris from the alveoli including allergens, microorganisms and inorganic particulate matter. All three cell types release pro-inflammatory mediators and we have demonstrated that interplay between these cells plays a vital role in regulating the pulmonary immune response [20, 21].

Regarding efficacious use of inhaled nano-drugs, the drug must be delivered intracellularly, involving NP uptake into and possibly translocation across the cell. For others, appropriate reactivity and delivery at the cell surface membrane is the aim [9, 22, 23]. However, it is important to appreciate the exact cellular responses, to avoid unwanted effects such as cytotoxicity, inflammation and tissue injury and therefore to optimise treatment. We hypothesised that NP size and surface modification would crucially impact on these processes, and the induction of oxidative stress would be a biomarker of unwanted effects of nano-drugs. Therefore in this novel study, we have examined the effect of nano-size and surface chemistry/charge of model polystyrene latex NPs on oxidative stress and cellular toxicity with immortalised human AT1 (TT1), primary human AT2 and MAC cells, representing the first cellular targets of inhaled nano-drugs in the human respiratory unit.

There is no standard *in vitro* model of the alveolar epithelial barrier to study drug transport, pharmacokinetics and bioreactivity; for example many *in vitro* studies utilise the A549 adenocarcinoma cell line as a substitute for primary human alveolar epithelial type II cells [24–26], whilst others utilise the Calu-3 human bronchial epithelial cell line, also derived from a pulmonary adenocarcinoma, to investigate changes in barrier function of large airway epithelium [27, 28]. We believe it is also relevant to use cell lines derived from normal lung cells and primary cells [21]. Furthermore, it is not possible to isolate sufficient primary human alveolar type 1 epithelial cells (many of which do not survive the procedure), and there is no commercially available source, thus, we have generated a unique immortal human AT1-like cell line (TT1) [29] from their progenitor cells, primary human AT2 cells [30]. In parallel, we study freshly prepared human lung AT2 cells [30] and MACs, from the same pieces of human lung tissue with normal appearance, removed during surgery for lung cancer. We have used these models in the following studies of the interaction of 50 and 100 nm polystyrene latex NPs, unmodified (UNP) and also surface-modified with amine (ANP) and carboxyl groups (CNP).

## Results

### Assessment of particle size and surface charge of latex nanoparticles

The interaction of nanosized-materials with body fluids is an early event; we and others have shown that components of extracellular fluids adsorb to the particles [31–33]. Importantly, we recently showed that polystyrene latex nanoparticles adsorb components of the tissue culture medium [31], which is likely to alter the surface charge and format of the NPs presented to the cells. Here the hydrodynamic diameter and surface charge density of each surface group (Table 1) in distilled water (DW) and in tissue culture medium (DCCM1 and RPMI) were measured. All NPs were monodisperse in DW but formed small agglomerates in DCCM1 and RPMI (Table 1), as indicated by the increase in average hydrodynamic diameters and polydispersity index values (PDI), likely reflecting adsorption of proteins and other components of the medium [31]. The surface charge densities (measured as zeta potential; Table 1) of the NPs also depended on the dispersing medium; the 50 nm ANP (+43.7 ± 1 mV), CNP (−46.7 ± 1.27 mV) and UNP (−50.5 ± 2.56 mV) show strong positive and negative surface charge densities in DW relating to their surface functional group (−OH for UNP,-COOH for CNP and –NH2 for ANP; Table 1), but their surface charge became very similarly moderately negative, regardless of their original charge, in DCCM1, as follows: ANP (−11.4 ± 0.90 mV), CNP (−13.1 ± 0.89 mV) and UNP (−15.5 ± 0.81 mV) and in RPMI ANP (−35.2 ± 0.56 mV), CNP (−33.0 ± 0.97 mV) and UNP (−14.8 ± 2.10 mV).

**Table 1:** Surface chemistry, hydrodynamic diameter, and surface charge density of unmodified- (UNP), carboxyl-modified (CNP) and amine-modified (ANP) latex nanoparticles in distilled water (DW) and tissue culture medium (DCCM1 and RPMI)

Particles	Surface functional group	Average diameter nm(±SD)	Polydispersity index	Zeta potential mv(±SD)



The data are presented as mean ± standard deviation (SD), *n* = 3^β^

^β^Nanoparticles were suspended in distilled water and DCCM1 or RPMI at a concentration of 10 μg/ml and bath sonicated for 2 min before measurement (see methods)

### Effect of size and surface chemistry on cell viability

**Fig. 1:**
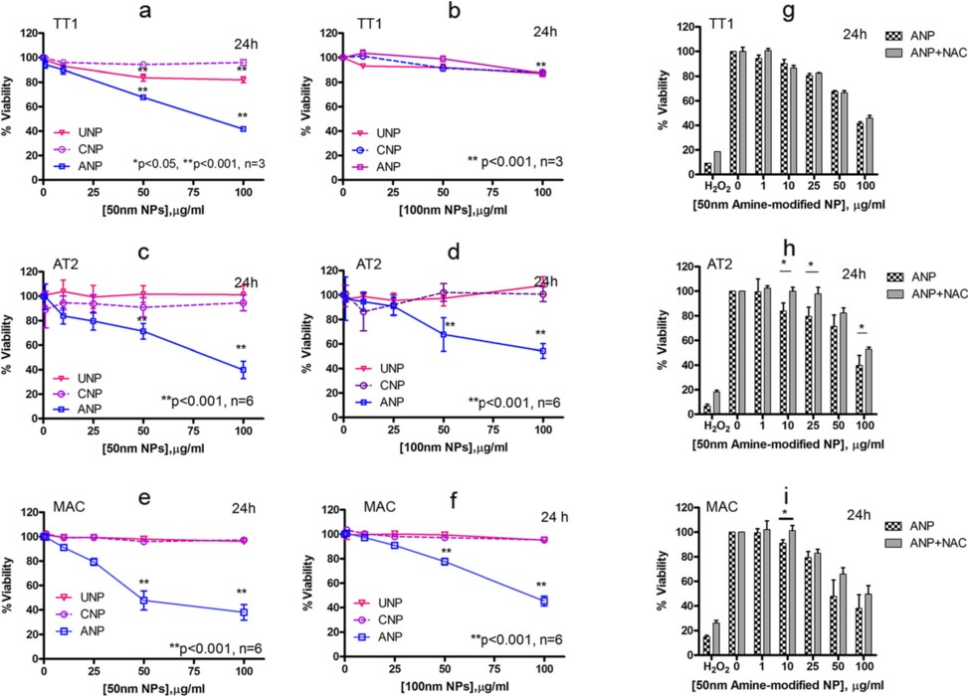
Viability of TT1, AT2 and MAC following 24 h exposure to 50 nm and 100 nm polystyrene nanoparticles. 50 nm ANPs, at 50-100 μg/ml, caused significant cell death in all cell types (**a**, **c**, **e**). 100 nm ANP, 50 and 100 μg/ml, caused significant cell death in AT2 and MAC but not TT1 cells (**b**, **d**, **f**). Addition of antioxidant N-acetylcysteine (NAC), 10 mM, did not prevent TT1 cell death (**g**), whereas NAC protected AT2 and MAC from the effects of low concentrations of ANPs (**h**-**i**). UNP and CNP had no effect on cell viability. *p < 0.05, **p < 0.001 and *n* = 3 TT1 replicates and *n* = 6 subject samples for AT2 and MAC

Previously we reported that 50 nm ANP induced cell death and pore formation at the cell membrane of TT1 cells, together with increased release of IL-6 and IL-8 and activation of caspase 3/7 and 9 [31]. Here, we further investigated the effect of NPs on their ability to induce intracellular reactive oxygen species (ROS) as this might impact on cell viability and bioreactivity. The 50 nm ANPs were reported to be more toxic than 100 nm ANPs; 50 nm ANPs were also more toxic than 50 nm UNPs and CNPs after 24 h exposure [31]. In this study, we showed that following 4 h exposure, there was only a slight toxic effect of ANP on TT1 at concentrations between 50-100 μg/ml (Additional file 1: Figure S1). We also investigated if the same response profile would occur with AT2 and MACs using a similar NP concentration range and 24 hour exposure. The cytotoxicity of 50 and 100 nm NPs against TT1, AT2 cells and MACs showed a similar pattern of response, where ANPs, but not UNPs or CNPs, were very cytotoxic (Fig. 1a-f). The 50 nm ANPs were toxic even at the lower concentrations of 10 and 25 μg/ml (*t* = 24 h, *n* = 6), inducing approximately 20 % cell death, compared to <10 % cell death for the same concentrations of 50 nm UNP and CNP and 100 nm ANPs. ANPs, 50 μg/ml, induced approximately 50 % cell death in MACs, compared to 30−35 % cell death (*t* = 24 h, *n* = 6) in the epithelial cells, although the highest concentration, 100 μg/ml ANP caused a similar degree of cell death in all cell types (~60 %; Fig. 1b, d and f). At the highest concentration of 100 nm NPs there was approximately 45−50 % AT2 and MAC cell death (Fig. 1d and f), although TT1 cells exhibited less than 20 % cell death at this concentration (Fig. 1b). We suspected that oxidative stress might associate with ANP toxicity, and therefore, the addition of antioxidant N-acetyl-cysteine (NAC, 10 mM) would reduce cell death following ANP exposure. When TT1, AT2 cells and MACs were exposed to the 50 nm NPs (*t* = 24 h) in the presence of NAC (Fig. 1g-i, Additional file 1: Figure S2), there was little effect on the UNP- or CNP-treated cells, as expected due to little effect of the NPs alone (Additional file 1: Figure S2). Regarding the marked cell death induced by 50 nm ANP, there was no improvement in cell viability of TT1 cells (Fig. 1g). In MACs, NAC had only a small protective effect on cell death at 10 μg/ml ANP (Fig. 1i). However, in AT2 cells (Fig. 1h), NAC prevented the effects of 10 and 25 μg/ml 50 nm ANP, and caused a small improvement in the effect of 100 μg/ml ANP on AT2 cell viability.

We also observed toxicity of NPs in all cell types using the lactate dehydrogenase (LDH) assay to examine membrane integrity (*n* = 6, *t* = 4 and 24 h; Additional file 1: Figure S3–S4). We previously observed a marked release of LDH by TT1 cells exposed to 50 nm ANP (but not UNP or CNP) which paralleled the formation of “holes” within the cell membrane [31]. AT2 cells exposed to all types of 50 and 100 nm ANPs released LDH (Additional file 1: Figure S3); the most significant release of LDH was on exposure to 50 nm CNPs, ANPs and 100 nm ANPs (*p* < 0.001, *n* = 6). The release of LDH was time and NP concentration dependent; the smaller NPs induced the highest LDH. At the higher concentrations of 50 nm ANPs (25-100 μg/ml), there was up to 60 % LHD release, which mirrored MTT estimation of AT2 cell death (Fig. 1c-d). In contrast, in the MAC studies, the 100 nm NPs had the greatest effect on LDH release compared to 50 nm NPs following 24 h exposure (*p* < 0.001, *n* = 6; Additional file 1: Figure S4), which was also concentration and time dependent. ANPs exhibited higher toxicity than UNPs and CNPs, and there was marked release of LDH at the higher concentrations of 100 nm ANPs (25-100 μg/ml) causing up to 75 % LDH release following 24 h exposure, in parallel to the MTT measure of cell death (Fig. 1e and f). Interestingly, LDH did not exactly mirror MTT measure of cell death for MAC experiments exposed to 100 nm UNP and CNP.

### Effect of nanoparticles on the release of inflammatory mediators, IL-6 and IL-8

Following 24 h exposure, all types of NPs activated significant increased release of IL-6 and IL-8 (*p* < 0.001, *n* = 6) by AT2 cells and MAC (Additional file 1: Figure S5). The increase in IL-6 release by AT2 was much the same regardless of NP concentration or surface modification. This may in part reflect the similarity in surface charge density (zeta potential) between the NPs, which was similar in DCCM1 medium, between −11.4 and −15.5 mV (Table 1), but does not address the lack of effect of increasing NP concentration. The marked increase at even low NP concentrations may be due to induction of the maximal IL-6 response. Neither is it clear why there are no differences between the magnitude of the effect of 50 and 100 nm NPs, considering the marked increase in NP numbers and surface area/unit weight of the 50 nm NPs, as discussed previously [31]. In contrast, an effect of particle size was observed for release of IL-8 (Additional file 1: Figure S5d-f); 50 nm NPs induced a significantly greater release of IL-8 than that of the 100 nm NPs. However, as for IL-6, there was no effect of increasing NP concentration on AT2 cell IL-8 release. In contrast, for MACs (Additional file 1: Figure S6) increased release of IL-6 and IL-8 correlated with NP concentration. Surface chemistry was important (*p* < 0.001, *n* = 6); ANPs and UNPs induced the greatest, similar levels of IL-6 and IL-8 release, compared to CNPs. Again, there was little effect of NP size on IL-6 release, whereas 100 nm UNP and CNP induced more IL-8 release than did the 50 nm NPs. We previously reported that all three types of 50 nm NPs stimulated a concentration-dependent release of IL-6 and IL-8 by TT1 cells [31]. Others have shown that polystyrene latex stimulated IL-8 release by A549 cells; the smallest, 60 nm NPs, caused the highest release compared to 200 and 500 nm NPs [34]. Prietl et al. reported a similar pattern of IL-8 release by macrophages exposed to 20, 500 and 1000 nm carboxyl modified latex particles [35].

### Effect of nanoparticles on the activation of intracellular reactive oxygen species (ROS)

**Fig. 2:**
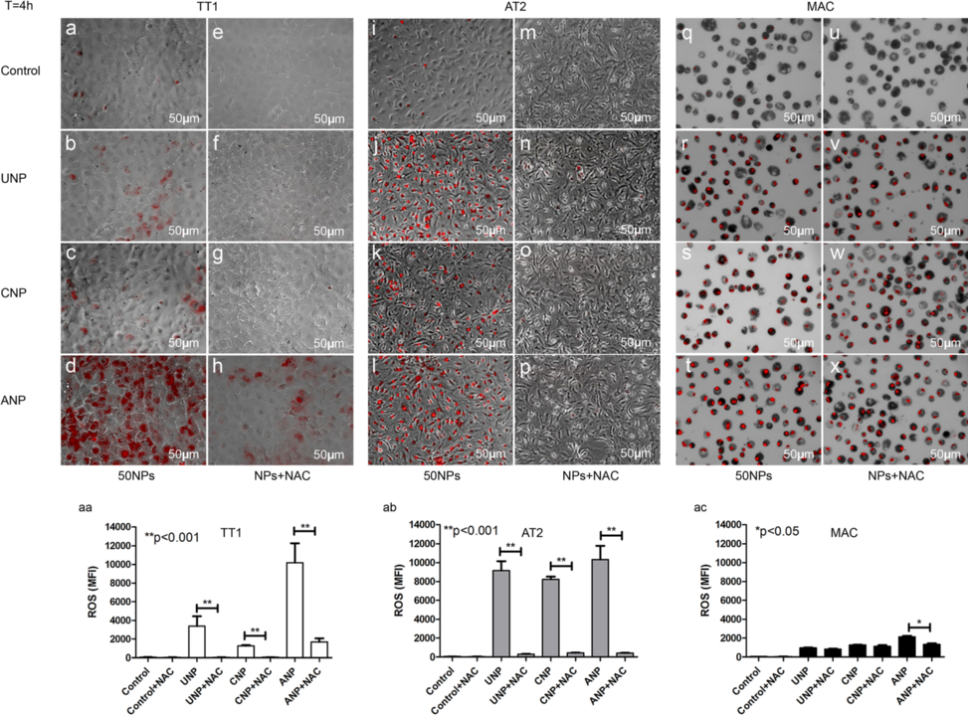
Induction of reactive oxygen species by 50 nm UNP, CNP and ANP in the absence and presence of antioxidant N-acetylcysteine. Cells were exposed to 25 μg/ml of 50 nm NPs for 4 h, alone (**a**-**d**, **i**-**l**, **q**-**t**) and in the presence (**e**-**h**, **m**-**p**, **u**-**x**) of 10 mM NAC. All types of NPs induced significant ROS production in AT2 (**i-l**) and MAC (**q**-**t**), whereas TT1 cells (**a**-**d**) were most susceptible to ANP. NAC treatment significantly prevented NP-induced ROS in epithelial cells (aa, **e**-**h**, ab, **m**-**p**), but not macrophages (ac, **u-x**). ROS were measured in live cells and data are presented as mean fluorescence intensity (MFI) ± SD (*n* = 3 TT1 replicates and 6 subject samples for AT2 and MAC) in aa-ac *p < 0.05; **p < 0.001

We used 2′, 7′-dichlorodihydrofluorescein diacetate (H2-DCFDA; to detect peroxide and singlet oxygen) and dihydroethidium (DHE; to detect superoxide radicals) dyes to monitor intracellular ROS. Both dyes indicated a similar pattern of ROS induction within TT1 cells; however, only DHE effectively detected ROS in AT2 and MAC, indicating differences in ROS production between the cells. ANPs (50 nm) significantly initiated ROS production in TT1 cells in a concentration dependent manner at 4 h, when there was no cell death (*p* < 0.001, *n* = 3; Additional file 1: Figure S1, S7a-d, s). The ROS detected by H2-DCFDA remained over 24 h, when there was significant cell death, as can be seen by cell loss in Additional file 1: Figure S7f. UNPs did not initiate ROS detected by H2-DCFDA in TT1, whereas CNPs took up to 24 h to induce ROS (Additional file 1: Figure S7). ANPs induced massive production of ROS (detected by DHE and H2-DCFDA) in TT1 cells, *p* < 0.001, *n* = 3, at both 4 and 24 h (Fig. 2d, aa. Additional file 1: Figure S7a-d, f, s-t). Although UNPs and CNPs induced TT1 ROS, it was much lower than that observed with ANP (Fig. 2b and c, aa; Additional file 1: Figure S7g-h, t). In contrast, all types of NPs initiated significant production of ROS (detected by DHE) in AT2 (*p* < 0.001, *n* = 3, Fig. 2j, k, l and ab) and MACs (Fig. 2r, s, t and ac), which in AT2 cells was concentration-dependent (Additional file 1: Figure S7i-r, u). This effect was completely eliminated in AT2 cells by co-incubating the antioxidant N-acetyl-cysteine (10 mM) with the NPs (Fig. 2n, o, p and ab), which also prevented ROS formation in TT1 cells exposed to UNP and CNP (Fig. 2f- g, aa), though only partially eliminated ROS in TT1 cells exposed to ANP (Fig. 2h, aa). In contrast, NAC had little effect when added to NP-exposed MACs, regardless of surface modification (Fig. 2v-x, ac). Although the induction of oxidative stress was observed following NP exposure to all types of NPs, differences were observed in the magnitude and profile of ROS activity relating to both surface modification and cell type (Fig. 2aa, ab, ac). Although the cells were seeded at the same density, in the case of the primary MACs not all the seeded cells adhered to the plate and the final number of MACs was less than those for AT2 and TT1 cells. This resulted in an overall reduction in the measured intensity of ROS in MAC (Fig. 2ac) compared with TT1 and AT2 cells (Fig. 2aa and ab).

### Effect of nanoparticles on glutathione flux

**Fig. 3:**
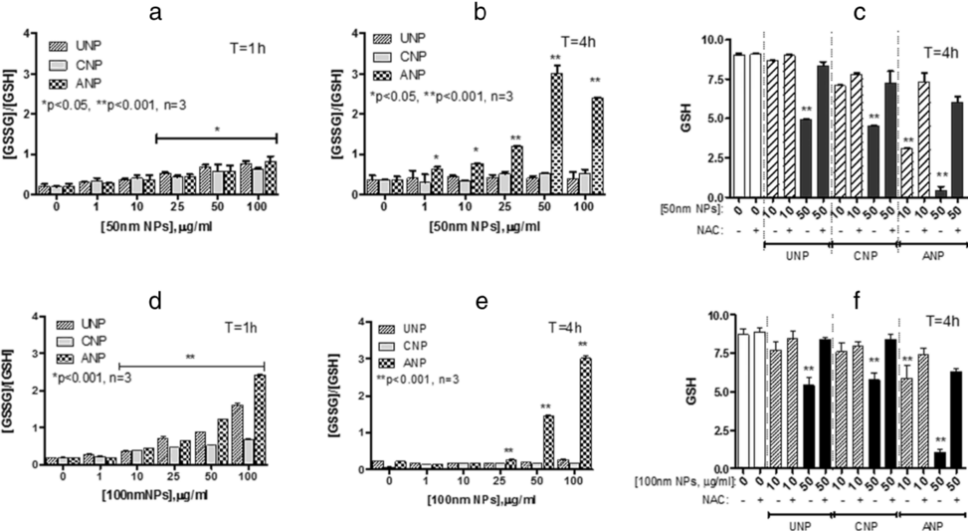
Effect of polystyrene nanoparticles on total cellular glutathione and TT1 cellular oxidised glutathione: reduced glutathione ratio (GSSG: GSH) at 1 and 4 h exposure. GSSG:GSH ratio following exposure to 50 nm (**a**, **b**) and 100 nm (**d**, **e**) NPs for 1 (**a**, **d**) and 4 h (**b**, **e**) respectively. Total GSH following exposure to 50 nm (**c**) and 100 nm (**f**) for 4 h with and without antioxidant N-acetylcysteine (NAC, 10 mM). ANPs caused the most significant increases in GSSG:GSH ratio, regardless of particle size. All three NPs caused a reduction in total cellular GSH, though this was most marked following ANP exposure (**c**, **f**). NAC treatment significantly prevented this effect (**c**, **f**). *p < 0.05, p** < 0.001; *n* = 3 replicates

As we have limited numbers of primary AT2 and MAC cells, we used TT1 cells as a model to study the effect of oxidative stress on glutathione flux. Cellular glutathione levels (GSH and GSSG) were measured in TT1 cells at 1 and 4 h after 50 nm NP exposure (Fig. 3a, b). After 1 h, the GSSG/GSH ratio (indicating the ratio of oxidised GSSG to reduced GSH) was increased, 2-3-fold above non-treated control cells, for all types of NPs (at concentrations of 50 and 100 μg/ml) reflecting oxidative stress (Fig. 3a-b). By 4 h (Fig. 3b), control (baseline) TT1 cell GSSG/GSH ratio had increased above that observed at 1 h. There was a further, significant increase in the GSSG/GSH ratio following ANP exposure, even at the lowest concentration of 1 μg/ml (1.5-fold control; *p* < 0.05, *n* = 3), which increased in a concentration dependent manner, reaching >6-fold that of unexposed cells (*p* < 0.001, *n* = 3) at 50 μg/ml ANP; this effect was not observed with UNP or CNP. Co-incubation of NPs with NAC for 4 h prevented the reduction of total cellular glutathione (GSH and GSSG combined; Fig. 3c). The highly significant fall (down to ~10 % of control, *p* < 0.001, *n* = 3) in glutathione following exposure to 50 nm ANPs could be markedly prevented by NAC treatment (down to ~67 % of control). A similar trend was also observed in TT1 cells exposed to 100 nm NPs, although the effect of all three NPs on increased GSSG/GSH ratio at 1 h was more noticeable than that seen following exposure to the 50 nm NPs at the same time interval (Fig. 3d). Remarkably, this effect disappeared for UNP and CNP at 4 h, but remained at very similar levels for the ANP-exposed cells (Fig. 3e). NAC prevented the reduction of cellular glutathione activated by 100 nm NPs (Fig. 3f) to a very similar extent to that seen with 50 nm NPs.

### Effect of nanoparticles on mitochondrial function and structure

**Fig. 4:**
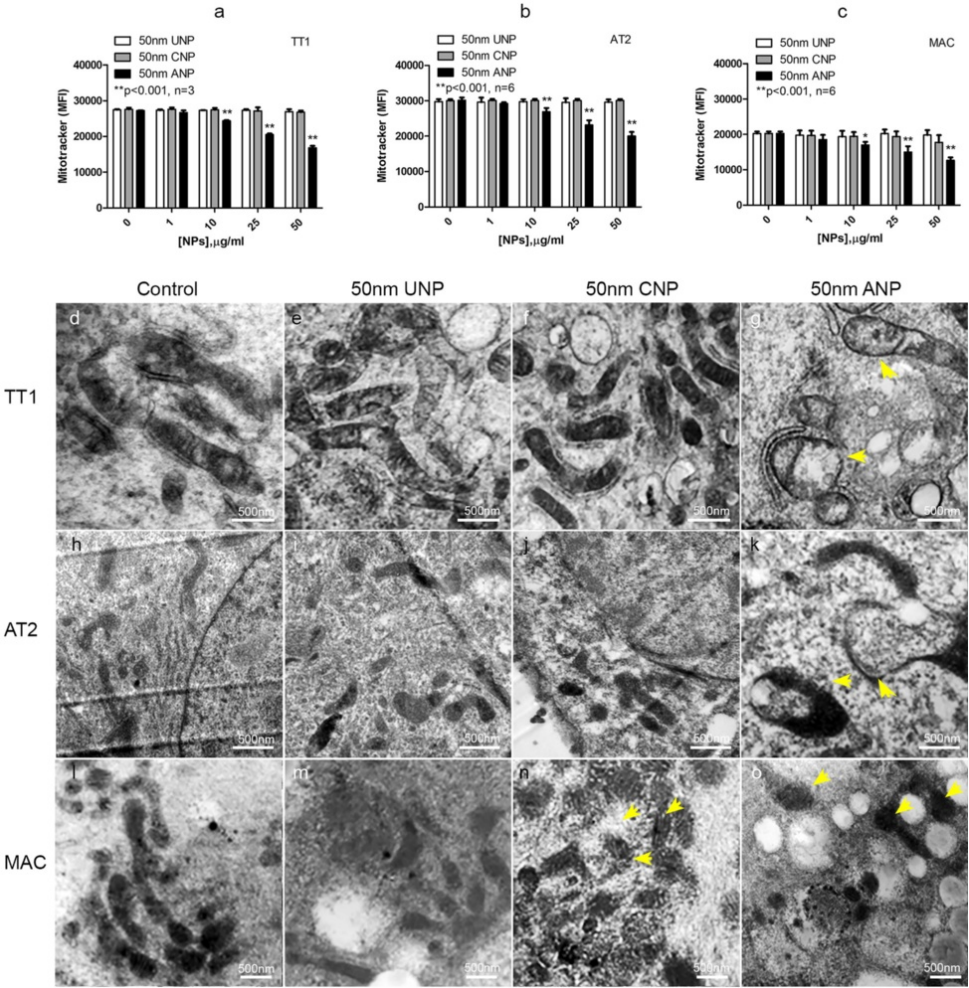
Effect of 50 nm polystyrene nanoparticles on mitochondrial membrane potential (**a**-**c**) and structure (**d**-**o**) following 4 h exposure**.** ANPs caused a significant reduction in the mitochondrial membrane potential (**p < 0.001, *n* = 3 TT1 replicates and 6 subject samples for AT2 and MAC) of all cell types (**a**-**c**) and altered mitochondrial structures by causing mitochondrial swelling (arrows in **g**, **k**, **o**) compared with the control (**d**, **h**, **l**). There was only slight mitochondrial swelling in MACs following CNP exposure (arrows **n**) not seen following UNP exposure (m). The number of total observed cells analysed/sample was 60 (*n* = 60); scale bar 500nm

**Fig. 5:**
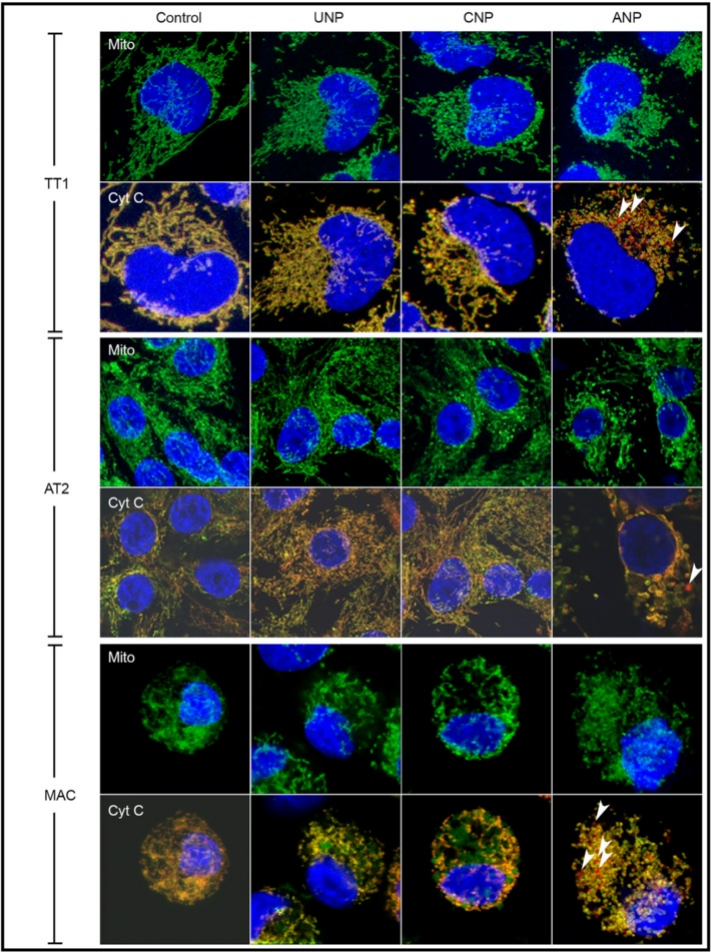
Effect of polystyrene nanoparticles on cytochrome C (Cyt C) release and the mitochondrial network (Mito) in TT1, AT2 and MAC. Exposure to 50 μg/ml 50 nm UNP and CNP had no effect on the release of Cyt C or the mitochondrial network. ANP caused disruption of the mitochondrial network (arrows indicate breakdown of Mitochondria in green) and initiated the release of Cyt C (arrows indicate the red area of Cyt C release) in all cell types). Cell nuclei, mitochondrial networks and cytochrome C are stained blue, green and red, respectively; *n* = 45 cells analysed/sample

Mitochondrial membrane potential and mitochondrial structure of TT1 cells following NP exposure (Fig. 4–5) were observed to compare with changes in ROS production, using MitoTracker® fluorescent probe, confocal microscopy and transmission electron microscopy (TEM). The MitoTracker® probe reflected mitochondrial membrane potential of the intact mitochondria; a significant decrease in mean fluorescence intensity (MFI), indicating reduction of mitochondrial membrane potential in all cell types exposed to 50 nm ANPs (*t* = 4 h, Fig. 4a-c; *p* < 0.001, *n* = 3 replicates TT1 and 6 subject samples AT2 and MAC). This was accompanied by mitochondrial swelling and disruption of the mitochondrial network in ANP-exposed cells (Fig. 4–5), as shown by TEM (Fig. 4, *n* = 60 observed cells) and confocal microscopy (Fig. 5, *n* = 45 observed cells). Mitochondrial swelling is a pathology of mitochondria indicated by an increase in volume of mitochondria due to the fluid influx as a result of altered mitochondrial membrane potential. The enlarged size of the mitochondria can be seen at the same magnification (same scale bar) as we show here in Fig. 4k, n and o, in comparison to the control cells in Fig. 4d, h and l. The structure of cristae collapse during the swelling process cannot be detected by the osmium contrast agent when using TEM. We also investigated changes in mitochondrial structure at the lower NP concentration range (1-25 μg/ml) using transmission electron microscopy (TEM) and did not see a difference compared to non-treated cells (data not shown). In the normal healthy cells the mitochondria form a network where each mitochondrion is linked to another as seen in control cells in Fig. 5 (the connected green fluorescent feature, control panel). Disconnection of the green fluorescent feature indicated the disconnected mitochondria within the network (change from green connect line to green dot), as seen following exposure to ANP (ANP, green fluorescence panel). Cytochrome C was stained with a red fluorescent signal and when co-localised with the mitochondrial fluorescent green signal, showed yellow. However, in ANP-exposed cells, cytochrome C is released from the mitochondria and this can be observed in a clear pure red fluorescent signal, indicating loss of mitochondrial integrity and apoptosis (arrows on the right ANP column in Fig. 5). Unlike TT1 cells, all NPs induced ROS production in AT2 cells, however only ANPs induced mitochondrial swelling and loss of mitochondrial membrane integrity, as seen by TEM (Fig. 4k), and breakdown of the mitochondrial network (Fig. 5). This was associated with the release of cytochrome C (Cyt C indicated with arrow) within the cells, though this was not as noticeable as that observed in TT1 cells (Fig. 5). Again, all types of NPs induced ROS in MACs; interestingly, unlike the epithelial cells, in MACs, both CNPs and ANPs initiated mitochondrial swelling (Fig. 4–5); however, breakdown of the mitochondrial network and release of Cyt C could only be observed in MACs exposed to ANPs (see arrows in Fig. 5).

### Uptake of nanoparticles by TT1, AT2 and MAC

**Fig. 6:**
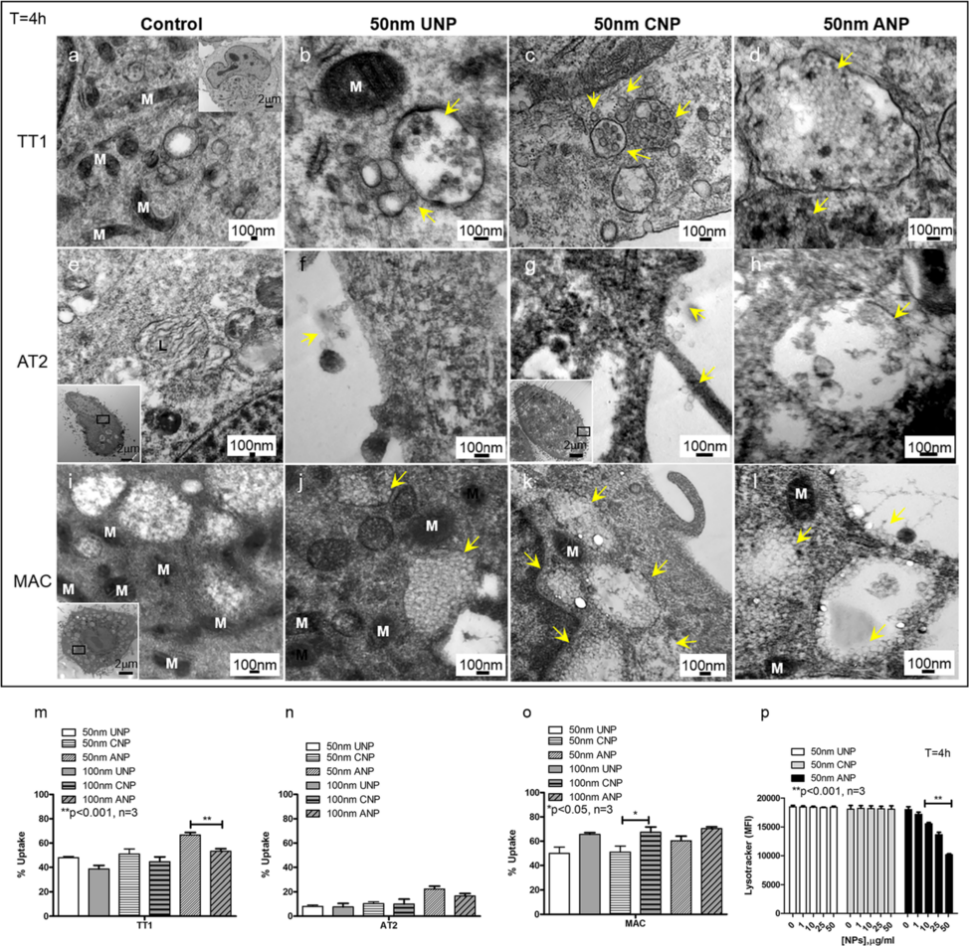
Transmission electron micrographs of TT1, AT2 and MAC following exposure to 50 nm UNP, CNP and ANP for 4 h. The data are presented as TEM images (**a**-**l**) and quantitatively as bar graphs (**m**-**o**). Following exposure to 50 μg/ml 50 nm NPs, TT1 and MAC internalised all three types of NPs, (**m**, **o**, arrows in **b**, **c**, **d**, **j**, **k**, **l**), whereas much lower numbers of AT2 cells internalised NPs (**n**). UNP and CNP were observed adhering to AT2 cell membranes (arrows in **f**-**g**). Once internalised, all types of NPs were observed mainly in endosomal structures. Percentage cell uptake is presented in m-o (*p < 0.05, **p < 0.001, *n* = 3, total 90 cells analysed). The reduction in mean fluorescence intensity (MFI) of Lysotracker® probe (**p**) indicates the lysosomal disruption following 4 h exposure of TT1 cells to ANPs (**p < 0.001, *n* = 3)

**Fig. 7:**
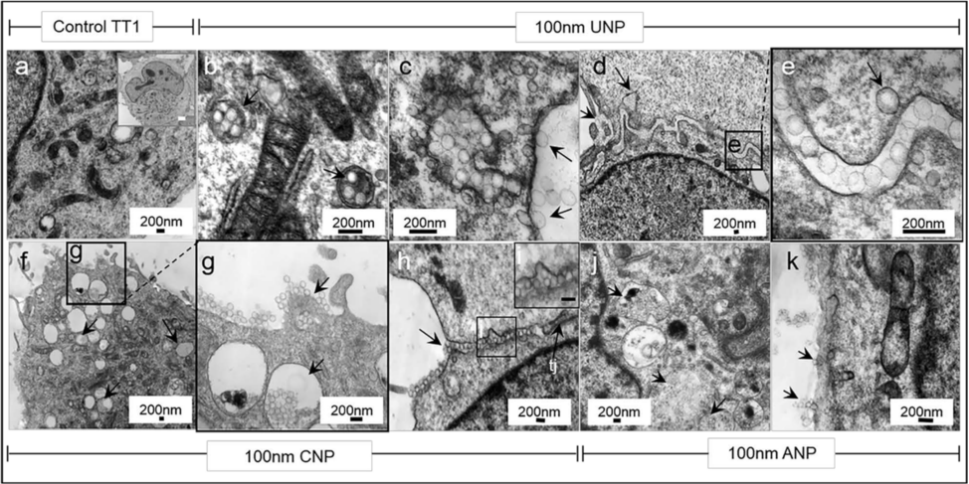
Interaction and uptake of 100 nm polystyrene UNP. CNP and ANP by TT1 cells 4 h after exposure. Following exposure to 50 μg/ml NPs, compared to non-exposed TT1 cells (**a**), UNPs were taken up *via* endocytosis as an agglomerate (arrows in **b**) and as individual particles (arrows in **c**). UNPs were also observed paracellularly and were taken up individually (arrows in **d**-**e**). Similar observations were made following TT1 cell exposure to CNPs (**f**; arrows in **g** indicate endocytosis and macropinocytosis). The CNPs also travelled paracellularly (left arrow in **h**, right arrow indicates tight junction-tj). The ANPs were taken up *via* endocytosis as agglomerates (arrows in **j**) and individually (arrows in **k**), but few were observed paracellularly. The percent cell uptake of all NPs by all cell types is shown in Fig. 6m-o in comparison with the 50 nm NPs. Scale bars in a-k are 200 nm; a total of 90 cells were examined

**Fig. 8:**
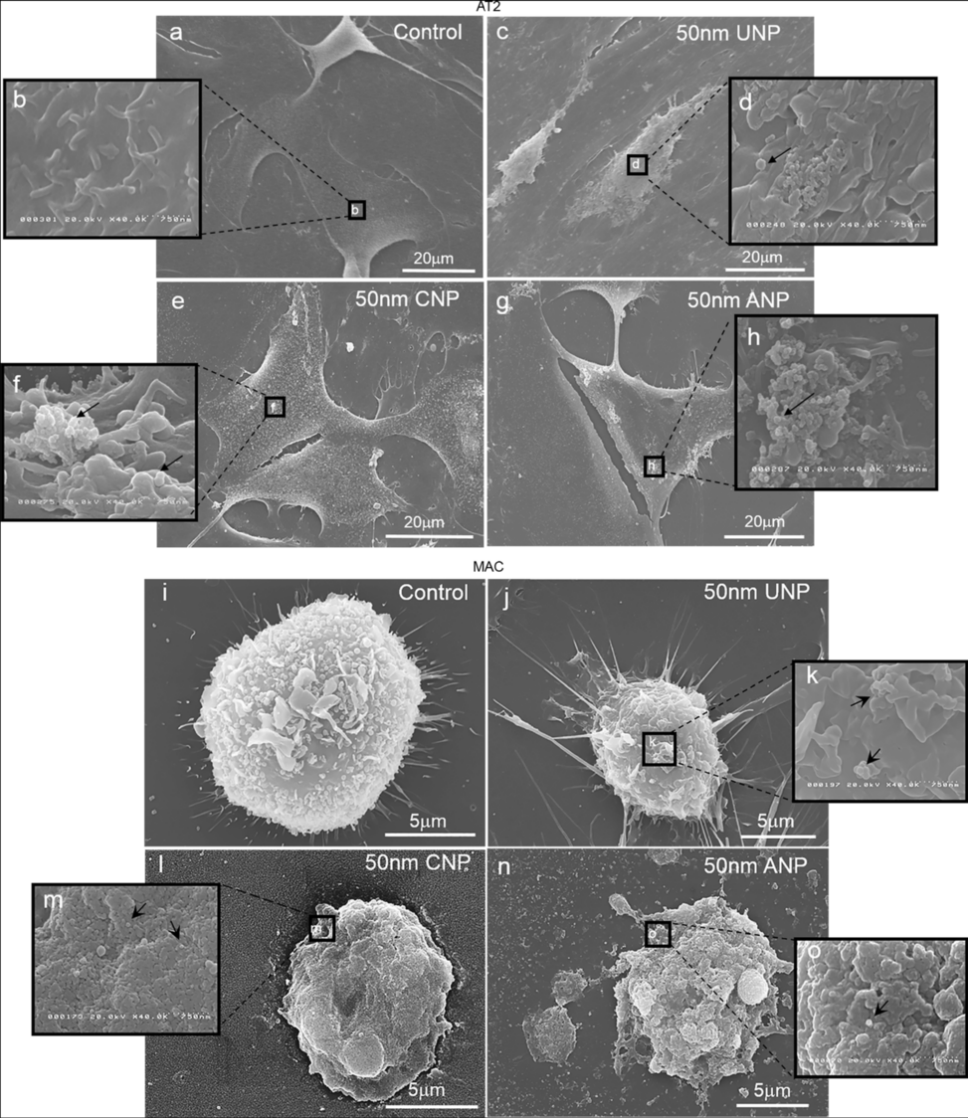
Interaction of UNP, CNP and ANP with the cell surface membrane of AT2 cells and MAC. AT2 cells exposed for 4 h to 50 μg/ml of 50 nm polystyrene UNP (**c**-**d**), CNP (**e**-**f**) and ANP (**g**-**h**) compared to the non-treated control AT2 (**a**-**b**). Both individual and aggregated forms of all types of NPs were observed on the cell surface membrane of AT2 cells, situated amongst microvilli (arrows in **d**, **f**, **h**; *n* = 60 cell observations). Scanning electron micrographs of control MAC (**i**) and MAC exposed to 50 μg/ml of 50 nm polystyrene UNP (**j**-**k**), CNP (**l**-**m**) and ANP (**n**-**o**) for 4 h. MACs were activated following exposure to UNP (**j**-**k**), CNP (**l**-**m**) and ANP (**n**-**o**). Both individual and aggregated forms of NPs were observed on the cell membrane of MACs (arrows in **k**, **m**, **o**). UNP triggered filopodia formation, while CNP and ANP initiated membrane blebbing. All type of NPs were observed in association with cell surface microvilli

TEM and scanning electron microscopy (SEM) were employed to observe nanoparticle-cell interactions and particle uptake (Fig. 6, 7 and 8). We recently showed that the uptake and transport of the same set of latex nanoparticles (*t* = 4 h) through TT1 cells involved both passive and active transport depending on their size and surface chemistry [16]. The 50 nm NPs largely entered TT1 cells *via* passive transport, while the 100 nm NPs entered mainly *via* clathrin- and caveolin-mediated endocytosis; 50 nm ANPs were internalised more rapidly than the UNPs and CNPs [16]. 3−8 % of 50 nm UNP and CNP translocated across the TT1 monolayer, without interfering with TT1 monolayer integrity [16]. It was demonstrated that the NPs can traverse between TT1 cells, until they reach a tight junction [16], also shown here in Fig. 7h and which suggests that the integrity of the tight junction (white tj, arrow), and its location, controls the translocation of these NPs between epithelial cells. The aim of the study did not include the effect of NPs on cell monolayer integrity and their translocation; however, work on the TT1 cell in this respect is described elsewhere [16]. In the current study, the cells were exposed to 50 μg/ml NPs, as this concentration exhibited very low toxicity (viability was 92−95 %, Additional file 1: Figure S1, [31]) at 4 h exposure and was a critical concentration at which a change in mitochondrial structure was observed. 40−60 % of TT1 cells and 50−70 % of MACs internalised NPs, whereas only 7−22 % of AT2 cells contained NPs (Fig. 6m-o). Interestingly, there was little difference between NP-functionalisation and the number of NPs taken up by each cell type, despite marked differences in cell viability, where ANP were most cytotoxic. Neither was there any difference between the particle sizes. This indicates that surface charge is an important component of the cytotoxic effect of the ANP, with the exception of TT1 cells, where 100 nm ANP-functionalised NPs caused relatively little cytotoxicity. The uptake of NPs by AT2 cells was much lower than that of the TT1 cells and the number of cytosolic NPs in AT2 cells was also much lower than that of the TT1 cells (data not shown). TT1 cells internalised all types of NPs following 4 h exposure (Fig. 6b-d, m, Fig. 7). The 50 nm and 100 nm NPs were observed within TT1 cell vesicles, suggesting active uptake (Fig. 6c-d, Fig. 7). Particles in endo/lysosomal compartments of TT1 were in the form of agglomerates, possibly aggregates (Fig. 6b-d). Cytoplasmic NPs were present as individual particles (Fig. 6b, c, l and Fig. 7c-e and k), suggesting passive uptake of NPs or that NPs might escape from endo/lysosomes. Use of the LysoTracker® fluorescent probe indicated an effect of NPs on lysosomal membrane integrity. The decrease in mean fluorescence intensity (MFI) of the probe indicated a decrease in the number of intact lysosomes within the cells following NP exposure. ANPs, but neither UNPs nor CNPs, caused a significant reduction in the MFI of LysoTracker® (*p* < 0.001, *n* = 3, Fig. 6p) suggesting that the amine-surface modified NPs precipitated lysosomal membrane damage and, subsequently, escaped to the cytoplasm possibly *via* a ‘proton sponge’ mechanism [36]. We previously reported that ANPs caused pore formation in the cell membrane which may be one mechanism of passive uptake of 50 nm ANPs [31]. In addition, NPs (Fig. 7c and k) appeared to adhere to the TT1 cell membrane and penetrate into the cell cytoplasm. The uptake of individual particles was also observed to occur at the lateral, paracellular and cell-cell interface, where NPs had tracked between the cells, up to the tight junction, before translocation as individual NPs across the cell membrane (Fig. 7d-e and h-i) as we previously reported [16]. It is difficult to assess whether particles that appear to be within the cytosol are membrane-bound. We used a sample preparation and staining technique, with osmium, uranyl acetate and lead citrate post-stain, to specifically identify membranes and believe that any membranes, including vesicular membranes should have been apparent. Thus, we believe that some particles appear to be free within the cytosol. And, importantly, this latter, paracellular process was less obvious with ANP suggesting different uptake mechanisms (Fig. 7j-k). In contrast to TT1 cells, most of which internalised all types of 50 nm NPs, only a small proportion of AT2 cells (6−20 % from Fig. 6, [16, 29]) were found to contain NPs (Fig. 6f-g, n). Intracellular ANPs were only found in the endosomal compartment (Fig. 6h), indicating active uptake.

To observe how NPs interact at the cell surface, we used scanning electron microscopy (SEM, Fig. 8) to observe the cell-nanoparticle interface. We previously reported that latex NP could initiate the protruding of microvilli in live cell experiments of TT1 interactions [31, 37] using scanning ion conductance microscopy [31]. A similar effect was observed here with AT2 (Fig. 8); the presence of all types of 50 nm NPs, 50 μg/ml, induced microvilli formation, possibly modifying NP interaction with AT2 cells, as the microvilli protruded and surrounded the NPs (Fig. 8a-d; [31]). Both agglomerated and individual NPs adhered to the cell membrane, amongst the cell surface microvilli, which were more dense and co-localised with the NPs (Fig. 8d, f and h). All types of 50 nm NPs were detected within the vesicle compartments of MACs, as agglomerates, suggesting phagocytic uptake (Fig. 6k, o). SEM indicated altered MAC morphology after exposure to NPs (Fig. 8i-o). UNP-induced changes in MAC morphology were of the classic activated macrophage, exhibiting extensive filopodia (Fig. 8j-k); in contrast, MACs exposed to CNP and ANP were devoid of filopodia and showed blebbing of the cell membrane (Fig. 8l-o), possibly prior to apoptotic cell death. All three NPs were found adhered to the cell membrane of MACs (Fig. 8k, m, o).

## Discussion

In this study, the response of human pulmonary alveolar epithelial cells and alveolar macrophages to NPs of different size (50 and 100 nm diameter) and surface modification (UNP, ANP and CNP, Table 1) were examined. Both size and surface charge are crucially important determinants of alveolar cell responses. In addition, the striking differences between cell types in their interactions with, and reactions to, nanoparticle exposure, are likely reflecting their functional role within the alveolus.

The ability of polystyrene latex NPs to induce cell death, cellular oxidative stress and oxidative damage were revealed. The increase in hydrodynamic diameters of all types of NPs in tissue culture medium DCCM1 indicated that these particles bind to proteins in the medium, mostly albumin, as described elsewhere [30, 31]. Thus the surface charge changed (zeta values) to be between −11 and -15 mV showing less difference between the different NP surface charges. Nevertheless, there were significant differences in the bioreactivity of these particles between the different cell types. One reason for this may be that albumin forms a soft corona that is easily displaced and, on interaction with specific components of the cell membrane [38, 31], the particles then exhibit their original surface chemistry. Although, the RPMI did not contain serum and protein, the aggregation or agglomeration of NPs could be observed with 100 nm NPs (all types) and 50 nm ANPs with zeta values between −16 and -27 mV. This suggested that salt and amino acids within RPMI could be involved in the aggregation or agglomeration. Interestingly, however, when IL-6 and IL-8 release (Additional file 1: Figure S5–S6) was examined in AT2 cells, the response was the same for all three particles, despite their surface chemistry. We recently showed that these NPs adsorb SP-D from human lung secretions [39], and we know that in this human AT2 cell model, surfactant is released [30]. We suggest that in this case, the albumin has been displaced by components of surfactant or other AT2 cell secretions that then triggers an identical response in the AT2 cells. We appreciate that it might be possible to avoid the effects of albumin adsorption from the medium by using air-liquid interface cultures and aerosolised particles [40]. Despite this, in this study we have shown markedly different responses between cells of the alveolar unit when challenged with NPs with different surface chemistry which we believe are important, and which we intend to study at an air liquid interface in future.

Nevertheless, the original surface charge of the polystyrene NPs was extremely important in their bioreactivity. As we and others have shown previously [16, 31, 41], amine-modified NPs are particularly cytotoxic at high concentrations [16, 17]. Even at lower, non-cytotoxic concentrations, 50 nm ANPs induced significant oxidative stress in all three cell types over a period of 24 h. Although UNP and CNP did not induce significant cell death, both these particles induced a high ROS response in AT2, similar to that observed following ANP exposure (Fig. 2; Additional file 1: Figure S7), but less so in TT1 cells, illustrating that induction of ROS varies according to the target cell. Addition of NAC significantly (p < 0.01, *n* = 3(TT1), 6(AT2)) reduced ROS production in TT1 and AT2 cells. MAC produced the lowest ROS levels both at baseline and following NP, likely due to the lower cell density of MACs compared to the TT1 and AT2 cells (Fig. 2). Therefore cells situated adjacently *in situ* responded quite differently to the same stimulus; furthermore, high ROS is not always linked to cell death. By using two types of dye tracking ROS, we found that ROS production by TT1 cells involved peroxide, singlet oxygen and superoxide (as both dyes exhibited a similar trend) whereas the ROS produced by AT2 and MAC was mainly superoxide. These findings indicate different oxidative stress processes between the cell types. The role of cationic NPs in induction of oxidative stress has previously been observed [17, 42, 43], using a variety of cell lines, both immune and structural cells. Induction of oxidative stress in a macrophage cell line by amine-modified NPs [17], amine-modified silicon nanoparticles (SiNP-NH_2_), resulted in cell death accompanied by ROS production [43] which was not observed with neutral and anionic silica nanoparticles. A similar phenomenon was described for the RAW 264.7, phagocytic cell line, exposed to amine-modified polystyrene nanoparticles [17], which lead to apoptotic cell death, in contrast to that in BEAS2-B cells, which exhibited necrotic cell death. However, in another study using cationic and anionic CdSe quantum dots [43], the cationic particles conferred the most toxicity against primary human airway epithelial cells, but this did not parallel ROS induction [44].

Increased TT1 cell ROS activation was accompanied by a reduction in total cellular glutathione and an increase in the ratio of oxidised glutathione to reduced glutathione (GSSG:GSH; Fig. 2, 3), particularly on exposure to 50 nm ANPs, accompanied by cell death, suggesting that increased ROS was the cause of cell death. Comparing the 50 and 100 nm NPs, the 50 nm NPs initiated a faster and higher ratio of [GSSG]: [GSH] which peaked at *t* = 30 min following exposure (data not shown), before falling at *t* = 1 h (Fig. 3); this then increased after 2 h exposure, peaking at 4 h (Fig. 3). A slower response was observed with TT1 cell exposed to 100 nm NPs which showed no effect at 30 min following NP exposure (data not shown) before the [GSSG]: [GSH] was increased markedly for all NPs at 1 h, above that for the 50 nm NPs, but which then fell for UNP and CNP, but for ANP peaked at *t* = 4 h exposure. This suggests a dynamic flux of [GSSG]:[GSH] which is temporal and related to particle size and charge. However, NAC treatment reduced oxidative stress but did not markedly ameliorate 50 nm ANP-induced TT1 cell death, again suggesting that induction of ROS is not always responsible for cell death. An intriguing discovery was that co-application of NAC could significantly reduce or prevent polystyrene-induced ROS production in epithelial cells, but had much less effect on ROS production by MACs (Fig. 2). This may be due to the low baseline of ROS production in MACs exposed to NPs, reflecting lower cell numbers and therefore reducing the sensitivity; a significant protective effect of NAC was only observed with MACs exposed to ANP (*p* < 0.05, *n* = 3). Xia et al. [17] reported that NAC could not protect RAW264.7 exposed to amine-modified polystyrene latex at 8 h, but this protective effect was re-observed at 16 h indicating time dependent process. NAC may protect cells because: i) NAC is a free radical scavenger and precursor of GSH and would be expected to protect GSH as well as replenish used GSH or supplement existing GSH; ii) NAC might also activate anti-apoptotic signal transduction pathways involved in cell survival [45, 46]. The inability to prevent polystyrene NP-induced ROS in MACs using NAC suggests that either the depletion of GSH was greater than that provided by supplementary NAC treatment, and/or that an alternative process is causing ROS production.

Nevertheless, ROS markedly increased in AT2 and MAC following exposure to all types of NPs indicating that induction of ROS was an important consequence of NP exposure, but supporting the proposal that other mechanisms are involved in cell death, which was largely a feature of ANP exposure. Previous studies indicate that early mitochondrial injury is an important feature of amine-modified polystyrene NP-induced cell death [42], where mitochondrial integrity and structure were affected. Thus, in a comparative study of the effects of amine-modified, 60 nm polystyrene particles on RAW 264.7 and BEAS2-B cell lines, the mitochondria were identified to be a target but the cellular reaction differed. Mitochondria in RAW 264.7 cells were swollen and lacked cristae exhibiting apoptotic cell death. In contrast, in BEAS2-B cells, the mitochondria were condensed and eventually disappeared exhibiting necrotic cell death. These differences between cellular responses to the same amine-modified nanoparticles were attributed to alternative modes of nanoparticle uptake, fate and bioreactivity within the cell. Mitochondria are considered to be the most important organelles in producing ROS and are also the first targets for ROS. It is possible that overproduction of ROS might damage the structure of mitochondria, triggering cell death *via* mitochondrial uncoupling and the release of cytochrome C and other apoptotic factors [47]. Indeed, in all three cell types, 50 nm ANP induced ROS, the swelling and breakdown of the mitochondrial network, and release of cytochrome C (Figs. 2, 4, 5), an important component of the apoptotic pathway. These findings are consistent with the increase in ROS and GSSG/GSH ratio in TT1 cells exposed to ANPs. This also corresponds with our previous observation that ANP exposure induced the release of TT1 cell caspase 9 and 3/7 suggesting that induction of the intrinsic apoptotic pathway is the major mechanism of ANP-induced TT1 cell cytotoxicity [31]. From our previous findings and this study, we have proposed a pathway of ANP-induced TT1 toxicity. Based on the findings in this study we suggest that this process may be applied with AT2 cells and MACs. However, we also observed LDH release, suggesting necrotic cell death, but which might have been due to secondary necrosis of apoptotic cells in the absence of efferocytosis and clearance of apoptotic cells. In contrast to these observations with ANPs, although UNP and CNP triggered increased ROS, notably in AT2 and MAC, this did not impact on mitochondrial integrity or cell viability, indicating the significance of surface chemistry in cellular bioreactivity.

As mentioned earlier, the cellular response to amine-modified particles is also believed to relate to cellular uptake mechanisms and quantity of particles internalised [35, 45–47]. Here, we studied the NP uptake of TT1 and another two cells types (AT2 and MACs) using TEM, an accurate but time consuming technique to quantify the percent of NP uptake. We previously employed other rapid techniques including flow cytometry, confocal microscopy, transwell plate studies to quantify particle uptake and transport across TT1 cells [16]. Our findings adds more information on NP uptake and help to complete the picture. All types of NPs were found in a high proportion of TT1 and MACs, whereas in AT2, only ANP were observed in a very low proportion of cells. One mechanism by which TT1 cells internalise molecules is by endocytosis, which may lead to transfer of molecules across the alveolar gas-blood barrier, *via* caveolae and clathrin coated vesicles [48, 49, 16]; the presence of 50 and 100 nm NPs in TT1 cells, as small and large agglomerates within endosomal vesicles, reflects this and suggests that another mechanism involves macropinocytosis. Cytoplasmic, individualised CNP and UNP could be due to passive uptake of the individual NPs (Fig. 7); these NPs may penetrate intracellular cell membranes or they might passively diffuse through cell membranes, without involving cellular energy. However, TEM cannot indicate whether the NPs are fusing with the phospholipid membrane or whether they are attached to it. Further investigation using high resolution TEM would be useful to establish this. Despite internalisation by TT1 cells, CNP and UNP were not overtly toxic. ANP were taken up apically by TT1 cells; cytoplasmic ANPs may reflect transfer *via* cell membrane pore formation, as demonstrated by us and others previously [31, 50]. We hypothesise that, at high concentrations, excessive membrane damage could cause cytotoxicity [51, 52]. Alternatively, release of ANPs due to endosomal rupture could occur [53, 54]; cytosolic ANPs in TT1 could initiate cell death, as suggested previously [31, 42].

Other studies describe the impact of cationic nanoparticle surface modification on uptake, which is consequently being used as a drug targeting strategy; cationic surface modification of nanoparticles, including poly(D,L-lactide-co-glycolide)/lipid-based nanoparticles, silica-titania hollow nanoparticles, iron nanoparticles, latex nanoparticles and megitoliposomes, enhanced their uptake by a wide range of cell lines [55–59]. However, endosomal and passive uptake of cationic nanoparticles may account for the observed toxicity of these particles, as described here, and elsewhere [17, 42]. A high density of amino surface groups on the surface of ANPs may trigger escape from endosomes and phagosomes by the so called ‘proton sponge’ phenomenon [37, 53, 54]. This could enhance the possibility of a direct interaction between ANPs and cell organelles, resulting in cell death. In contrast, for carboxyl-modified polystyrene nanoparticles, Fröhlich et al. [60] report that endosomal/lysosomal 20 nm carboxyl-modified nanoparticles within EAhy926 endothelial cell lines caused alteration of lysosomal enzyme activity but did not cause any lysosomal swelling and disruption. Such processes could explain the differences in cytotoxicity of neutral, cationic and anionic polystyrene in the present study. Thus, lysosomal disruption and release of potent lysosomal enzymes could contribute to ANP-induced cell death, as these enzymes have been shown to activate apoptotic cell death pathways, for example *via* direct damage to the mitochondria and proteolytic activation/inhibition of cellular processes.

## Conclusion

In this study of the response of three human cells that constitute the alveolar unit to 50 nm and 100 nm NPs, we have shown marked differences in the cellular responses depending on size and surface chemistry of NPs, and also on the type of cells. An important finding was that amine functionalization caused cytotoxicity in all three cell types, involving a process of induction of oxidative stress, mitochondrial disruption and Cyt C release, and likely leading to apoptotic cell death. Most of epithelial type 1 cells and macrophages internalised ANPs reflecting their functions in translocation of biomolecules across the gas–liquid interface and removal of organic and inorganic material from the alveoli, respectively. This implies that particle uptake is a pre-requisite to cell death, however, most AT2, largely secretory cells, showed much lower ANP internalisation, and yet ANPs were cytotoxic, indicating that there might be alternative cell surface-dependent mechanisms of cellular signalling that trigger oxidative stress and cytotoxicity. On the other hand, if cell death requires ANP uptake, followed by rapid death of the AT2 cells which contain the ANP, and survival of those that do not take up ANP (cell viability 70−80 % at ANP concentration of 50 μg/ml), this would confound the use of electron microscopy to relate ANP uptake to AT2 cell death because the dynamic response at cell-nanoparticle interface was observed. Further studies are required. Importantly, UNP and CNP did not induce marked cell death even though they were internalised by TT1 cells and MACs, and they induced ROS, mostly in AT2 cells and MACs. This suggests that the focus on induction of oxidative stress as a marker of cytotoxicity and therefore increased hazard to inhaled nanomaterials does not reflect the import of raised oxidative stress in protection from such challenges. Thus, NP uptake does not automatically relate to cytotoxicity, although induction of ROS suggests that other cellular processes might be harnessed. *In vivo*, the epithelial liquid lining layer contains significant concentrations of non-enzymatic antioxidants, which this study of NAC suggests would protect alveolar epithelial cells from NP-induced ROS. Type 1 cells cover 95 % of the alveolar surface and this unique study indicates that these cells are most robust in the presence of neutral and carboxyl-modified nanoparticles, which enter the cells. We suggest that type 1 cells provide an excellent target for inhaled NP drug delivery systems, both with the lung as a target, and for systemic delivery, being in close apposition to the microvasculature.

## Methods

### Culture of human immortalized alveolar epithelial type 1 cells (TT1)

The immortal TT1 cells were created from primary human alveolar epithelial type 2 cells isolated from the healthy region of tissues as previously described [29] which shows the same characteristics and phenotype as AT1 *in vivo* [29]. The TT1 cells were routinely grown in defined cell culture medium (DCCM1, Cadama, UK) supplemented with 10 % new-born calf serum (NCS) and 1 % penicillin/streptomycin/l-glutamine (PSG). They were seeded at a density of 0.5x10^6^ cells/well in 24-well plates until they reached confluence, within 2 days. 24 h prior to NP exposure, the cells were serum starved.

### Isolation of primary human alveolar macrophages (MAC)

The tissues used in this study were surplus tissue obtained following resection for lung carcinoma and written informed consent was obtained for all samples. The study was carried out with the approval of the Royal Brompton and Harefield Ethical Committee (Ref: 08/H0708/73). MAC were isolated from lung tissue as previously described [61]. Cells were isolated from a minimum of six different donors per experiment (*n* = 6). The lung tissues were perfused with 0.15 M sterile sodium chloride solution until the draining lavage became clear. The perfused saline was collected into 50 ml conical Falcon tubes and centrifuged at 1300 rpm (rotor radius is 168 mm) for 10 min at 20 °C. The cell pellet was re-suspended in serum-free RPMI culture medium supplemented with 1 % penicillin/streptomycin/l-glutamine (PSG) and plated onto 96 well culture plates at 0.2 × 10^6^ cells/well (and 0.5x10^5^ cells/well in 12-well plates for oxidative stress and uptake studies). These MACs settle down and adhere to the plate within 3 h of seeding. The medium was removed and the cells were was carefully rinsed with phosphate buffer solution (PBS) to remove the non-adherence cells. The cells were maintained in serum-free RPMI medium (0 % serum in RPMI) supplemented with 1 % PSG and incubated in 5 % CO_2_ at 37 °C.

### Isolation of primary human alveolar epithelial type 2 cells (AT2)

The tissue remaining following the MAC isolation was then used for AT2 cell isolation as previously described by Witherden et al. [62]. Cells were isolated from a minimum of six different subjects per experiment (*n* = 6). Cells were suspended in DCCM1 medium (Cadama, UK) supplemented with 10 % NCS and 1 % PSG and they were seeded on 96 well culture plates at 0.1 × 10^6^ cells/well (and 0.5x10^5^ cells/well in 12-well plate for oxidative stress and uptake studies). The wells were pre-coated with 1 % type I collagen solution (PureCol, Netherlands). Cells reached confluence 48 h after seeding, they were thoroughly characterised using electron microscopy illustrating their cuboidal morphology, lamellar bodies, tight junctions and microvilli [30, 62]. They were also stained positively for the AT2 cell marker alkaline phosphatase and expressed surfactant proteins A and C. Their retained the AT2 cell phenotype for up to 6 days [30, 62] and were used in these studies within three days of seeding. After reaching confluence, the cells were serum starved for 24 h before the exposure.

### Particle size and zeta potential

The 50 nm and 100 nm latex polystyrene nanoparticles (NPs), unmodified, carboxyl and amine-modified were purchased from Sigma Aldridge, UK. NPs were suspended in distilled water (DW) and DCCM1 culture medium without serum, (Cadama, UK) at a final concentrations of 10 mg/ml. NPs were vortexed and filtered through a 0.22 μm (50 nm NPs)/0.45 μm (100 nm) membrane filter. The samples were sonicated in a sonication water bath for 2 min just prior to measuring size and zeta potential using a Zetasizer Nano (Malvern Instruments Ltd, UK).

### Exposure of cells to NPs

MACs and TT1 cells were routinely seeded and cultured into each well of a 96-well or 24-well plates, in RPMI 1640 serum-free medium (MACs) and DCCM1 (TT1), 10 % new born calf serum (NCS), and 1 % PSG, respectively. AT2 cells were seeded at the same density on the collagen coated plate as described above. At confluence, 24 h prior to NP exposure, the medium of TT1 and AT2 was replaced with serum-free DCCM1. For MACs the cells were washed twice time to remove non-adhering cells and red blood cells before the exposure. The cells were exposed to 0–100 μg/ml (0–50 μg/cm^2^) NP for 4 and 24 h at 37 °C with or without N-acetyl cysteine (NAC), 10 mM, in serum free medium.

### Cell viability (MTT assay)

Following the 24-h exposure period, the medium was removed, the cells were thoroughly washed with PBS to remove residual NPs and then incubated with 3-(4, 5-Dimethylthiazol-2-yl)-2, 5-diphenyltetrazolium bromide (MTT) in fresh medium, 50 mg/ml, for 2.5 h at 37 °C. The medium was removed and 200 μl of DMSO was added to each well to dissolve the cells and the insoluble formazan dye. The plate was placed on a rotary shaker briefly and centrifuged at 14,000 g for 20 min to remove any residual NPs, before reading the optical density of the supernatants using a Thermomax microplate reader at 570 nm (MTX Lab Systems, USA). The viability of the cells exposed to NPs was then calculated as a percentage of the non-treated control cells (exposed to PBS). To determine the effect of NPs on the MTT assay, NPs were: (i) added to unexposed, control cells immediately before the assay, or (ii) added to the assay system following DMSO dissolution, and then processed identically to cellular experimental cells. The assay solutions were centrifuged at 14,000 g to remove the NPs from suspension prior to determination of the optical density of the supernatant, to avoid interference in optical density readings by NPs; experiments were carried out in triplicate for TT1 cells (*n* = 3) and *n* = 6 for the AT2 cells and MAC.

### Lactate dehydrogenase assay (LDH)

After 4-h and 24-h exposure periods, the conditioned medium was collected and centrifuged at 14,000 g for 20 min to remove the cell debris and residual NPs. LDH was analysed using the Cytotoxicity Detection kit PLUS (LDH; Roche, UK). For the positive control (100 % LDH release), 2 μl of cell lysis reagent (provided with the kit) was added into medium of non-treated cells and the cells were further incubated for 15 min before the medium, containing the LDH released from the lysed cells, was collected for centrifugation and LDH assay performed following the manufacturer’s protocol. 50 μl of the substrate (provided with the kit) was added to 50 μl of sample medium and the plates were incubated for 45 min before the stop solution was added. For the control and the substance control, a known concentration of LDH standard (0.05 U/ml) was incubated with a sample of DCCM1 medium, with or without increasing concentrations of the NPs and the assay performed in an identical manner. The data were collected by reading optical absorption at a wavelength of 492 nm using a Thermomax microplate reader (MTX Lab Systems, USA). There was no significant interference by the NPs because they were removed by centrifugation prior to reading the optical absorption, as described above. Three replicate experiments were performed for TT1 and six replicate experiments were performed for AT2 and MAC.

### Assessment of inflammatory mediator (IL-6 and IL-8) release

The release of inflammatory mediators interleukin 6 and 8 (IL-6 and IL-8) into the exposure media were measured using sandwich enzyme-linked immunosorbent assays (ELISA). The assays were performed using DuoSet® antibody kits (R&D systems, USA) following the manufacturer’s protocol. The potential nanoparticle interference was determined by adding NPs, 0–50 μg/ml, into standard solution of IL-6 or IL-8 before the ELISA was performed. No significant interference (*p* > 0.001, *n* = 3) was observed between the normal standard and the NP-addition standard suggesting that the NPs did not interfere with the ELISA assay. The data were collected by reading optical absorption at wavelength of 450 nm using a Thermomax microplate reader (MTX Lab Systems, USA). Six replicate experiments were carried out using cells isolated from six different donors.

### Reactive oxygen species (ROS)

Intracellular oxidative stress in cells was detected by imaging the fluorescence probes resulting from oxidation of dihydroethidium (DHE) or 2′, 7′-dichlorodihydrofluorescein diacetate (H2-DCFDA). The H2-DCFDA is a permeable compound which only detects intracellular ROS. DHE is a readily permeable fluorescent dye which can be oxidized by ROS, primarily superoxide, to yield ethidium molecules. Ethidium subsequently binds to DNA, which produces a detectable red fluorescence signal. After the exposure cells were washed twice with warm PBS and incubated with 200 μl of 10 μM DHE or 5 μM H2-DCFDA (both from Invitrogen, UK) in serum-free medium for 20 min. At the end of H2-DCFDA or DHE incubation, cells were washed twice to remove extracellular probe. The cells were imaged using Leica SP2 inverted fluorescent microscopy (Germany) using optical zoom ×10 and ×20. During imaging, the cells were maintained with 5 % CO_2_ and 95 % O_2_ in the live cell imaging chamber with heated base setting at 37 °C. The mean fluorescent intensity (MFI) of the H_2_DCFDA (ex488/em512nm) and DHE (ex535/610 nm) in the images was quantified using ImageJ (FIJI). Experiments were performed in triplicate.

### Glutathione assay (GSH)

Cellular antioxidant glutathione (GSH) was analysed using GSH/GSSH ratio assay kit (Calbiochem, Merckbioscience, UK) following the manufacturer’s protocol. Briefly, after the exposure cells were collected by centrifugation at 2000xg for 10 min at 4 °C. The cell pellet was sonicated in 1-2 ml of cold PBS buffer for 10 min and the cell debris was removed by centrifugation at 10,000xg for 15 min at 4 °C. The supernatant was collected and deprotonated by adding 10 % metaphosphoric acid and centrifuged at 5000xg for 10 min at 4 °C. The supernatant was stored at −20 °C until the assay was carried out. The optical absorbance at a wavelength of 450 nm was determined using a Thermomax microplate reader (MTX Lab Systems, USA). Experiments were carried out in triplicate.

### Monitoring mitochondrial pathology using MitoTracker® Green FM

MitoTracker® Red FM is a readily permeable fluorescent dye exhibiting potential-dependent accumulation in active mitochondria within live cells. The loss of mitochondrial membrane potential inside the cells will cause a reduction in the accumulation of the dye. Following NP exposure, the cells were washed twice with PBS before they were incubated with 50nM of MitoTracker® Red FM in RPMI medium for 45 min at 37 °C in 5 % CO_2_. At the end of the incubation, cells were washed twice with PBS to remove the excess probe before the fluorescent intensity of each well was measured using the Multi-detection multiplate reader SynergyTM HT (BioTek® Instrument Inc., USA) at an excitation and emission wavelength of 490 and 516 nm, respectively. Three and six replicate experiments were carried out on TT1 cells and AT2/MAC respectively.

### Monitoring lysosomal integrity using Lysotracker Red DND-99

The readily permeable probe, Lysotracker Red DND-99 (Invitrogen, UK), was used to monitor number of intact lysosomes following 4 and 24-h treatment with NPs. Following NP exposure, the cells were rinsed with PBS twice and incubated with RPMI culture medium containing 50 nM of the Lysotracker Red DND-99 probe, for 40 min. At the end of the incubation period, cells were washed twice with PBS to remove the excess probe and fluorescence intensity in each well was quantified using the Multi-detection multiplate reader SynergyTM HT (BioTek® Instrument Inc., USA) at excitation and emission wavelength of 577 and 590 nm, respectively. The experiments were performed in triplicate.

### Confocal fluorescence microscopy

For fluorescence microscopy, following NP exposure the cells were fixed with 3.5 % paraformaldehyde, and permeabilised with 0.1 % triton X-100 for 20 min. At least 20 cells were surveyed per one slide (*n* = 10) and three separate experiments were performed (total *n* = 30). Cells were next washed twice with PBS and blocked with 1 % BSA in PBS for 30 min before staining with Alexa fluor® 555 anti-cytochrome C (1:100 in blocking buffer; Invitrogen, UK), for 1 h. Cells were washed with PBS and stained with the mouse monoclonal primary antibody to mitochondria (Abcam, UK; dilution 1:100 in blocking solution) for 1 h. The cells were washed and stained with Alexa fluor488 anti-mouse IgG secondary antibody (1:100 in blocking buffer; BD Pharmingen, UK). Cells were rinsed well and mounted onto the slides with ProLong® Gold (Invitrogen) before they were visualised using Leica SP5 inverted confocal microscopy (Leica, Germany). Not less than 15 cells were observed in each slide and three slides were prepared for each sample, *n* = 45 cells (total observed).

### Transmission electron microscopy (TEM)

Following NP exposure, the cells were rinsed and fixed with 2.5 % glutaraldehyde for 2 h and then rinsed with sodium cacodylate buffer. The samples were post-fixed in 1 % osmium tetroxide for 1 h and dehydrated (with 25, 50, 75 % and dry ethanol) before embedding into resin (Araldite®) and microtomed into ultra-thin section (50−120 nm thick). The obtained sections were stained with 2 % uranyl acetate (*w*/*v* in ethanol) and lead citrate before viewing under TEM. Samples were processed for observation using TEM (Hitachi H7000 Nissei Sangyo Co., Ltd, Japan).

### TEM particle uptake analysis

This technique was adopted from our previous study [63]. Three embedded samples obtained from three replicate experiments were prepared, and three sections were cut from each embedded sample for viewing (total 9 viewing sections (grids) per one sample). At least 10 cells were randomly selected from one section for observation (total viewing 90 cells per sample). The percent cell uptake which was calculated from the number of particle-internalised cells against the number of total cells in each embedded sample (30 cells/3 viewing sections), and the percent cell uptake was calculated from 3 replicate experiments (*n* = 3) to achieve the total number of 90 observed cells (from 9 viewing sections).

### Observation of mitochondrial structure

Three embedded samples obtained from three deprecated experiments were prepared, and two sections were cut from each embedded sample for viewing (total 6 viewing sections (grids) per one sample). At least 10 cells were randomly selected from one viewing section, and their mitochondrial structures were observed (total viewing *n* = 60 cells per sample).

### Scanning electron microscopy (SEM)

For SEM analysis, cells were seeded onto coverslips for TT1 cells and MAC, and on collagen coated coverslips for AT2 cells. After NP exposure, the cells were processed using glutaraldehyde fixative (2.5 %) for 2 h and osmocated for 1 h. The coverslips were then critical point dried by immersion in hexamethydisilazane (HMD) before attaching to the stubs. The specimens (*n* = 60; 20 cells were randomly recruited from three separate experiments) were coated with gold before being visualised under Hitachi S4000 SEM microscopy (Nissei Sanyo, Japan).

### Statistical analysis

The data are presented as mean ± standard deviation (SD) where three and six replicate experiments were performed. The data were analysed by one-way and two-way analysis of variance (ANOVA) with Post-hoc analysis (Bonferroni) using GraphPad Prism 5 software. Differences were considered significant at *p < 0.05, **p < 0.001 and ***p < 0.0001. The number of experiments have been described for each assay above and as appropriate.

## Additional file

Additional file 1: Figure S1. Viability of TT1, AT2 and MAC following exposure to 50 nm NPs for 4 h. Low toxicity was observed in TT1 and MAC exposed to high concentrations of ANPs at *t* = 4 h. At the low concentrations of 1–25 μg/ml ANPs did not show significant toxicity in any cell type. **p* < 0.05, *n* = 3 replicates TT1, and 6 subject samples AT2 and MACs. **Figure S2.** Effect of adding the antioxidant N-acetyl cysteine to the 50 nm nanoparticles on cell viability. TT1 (a-b), AT2 (c-d) and MAC (e-f) were exposed to 50 μg/ml of 50 nm UNP (a, c and e) and CNP (b, d and f). There was no cytotoxicity on exposure to the NPs, neither was there a significant effect of NAC. *n* = 3 replicates TT1 and 6 subject samples AT2 and MAC. **Figure S3.** Release of lactate dehydrogenase following exposure of AT2 cells to 50 and 100 nm UNPs, CNPs and ANPs. The data are expressed as percent of the total cellular LDH in the unexposed control cells. The 50 nm NPs caused release of LDH which was significantly increased relative to NP concentration on exposure to CNP and ANP (b-c; ***p* < 0.001, *n* = 6 subject samples). Exposure to 100 nm UNP and CNP had no effect on LDH release (d-e), whereas 100 nm ANP induced significant release of LDH in a concentration dependent manner (f). The 50 nm ANP induced the highest level of LDH release compared to all other NPs. C, ***p* < 0.001, *n* = 6 subject samples. **Figure S4.** Release of lactate dehydrogenase following exposure of MACs to 50 and 100 nm UNPs, CNPs and ANPs. There was significant, NP concentration dependent release of LDH by MACs following exposure to all 100 nm NPs (d-f), which was most significant following ANP (f; ***p* < 0.001, *n* = 6 subject samples). Although all types of 50 nm NPs caused some release of LDH, this was only significant for ANP (c); the response was NP concentration dependent but did not reach the same level as that following 100 ANP exposure (c, f; ***p* < 0.001, *n* = 6 subject samples). **Figure S5.** Release of IL-6 and IL-8 following exposure of AT2 cells to UNPs, CNPs and ANPs. Both 50 and 100 nm NPs caused significant release of IL-6 and IL-8 (***p* < 0.001, *n* = 6 subject samples), although not in a dose dependent manner. There was no difference in level of the IL-6 release between the different types of NPs. **Figure S6.** Release of IL-6 and IL-8 following exposure of MACs UNPs, CNPs and ANPs. Both 50 and 100 nm NPs caused a significant, dose dependent increase in IL-6 and IL-8 release from MACs (***p* < 0.001, *n* = 6 subject samples). Although there was no difference between 50 and 100 nm NP on IL-6 release (a-c), the 100 nm NPs caused more IL-8 release than 50 nm NPs. d-f, ***p* < 0.001, *n* = 6 subject samples. **Figure S7.** Induction of reactive oxygen species in TT1 and AT2 cells exposed to UNP, CNP and ANP. The data are shown as images (a-r) and quantitatively as bar charts (s-u). ANP initiated ROS production following 4 h exposure at concentrations of 25 μg/ml and above (b-d; s). ROS activated by 50 μg/ml of ANPs remained significantly high at 24 h (f, t), CNP took up to 24 h to initiate ROS production in TT1 cells (g; t), although this was lower than that for ANP. UNPs initiated only a small, but significant degree of TT1 cell ROS production at 24 h (h; t). All types of NPs caused increased AT2 cell ROS production in a concentration dependent manner (i-r; u). The fluorescence intensity of ROS are quantified and presented as mean fluorescence intensity (MFI ± SD) as shown in s-u. ***p* < 0.001, ****p* < 0.0001, *n* = 3 replicate experiments for TT1 cells and 3 subject samples for AT2 cells.

## Abbreviations

AT1: Human alveolar epithelial type 1 cells
TT1: Immortalised human alveolar type 1 cells
AT2: Primary human alveolar epithelial type 2 cells
ANP: Amine-modifed polystyrene latex nanoparticles
CNP: Carboxyl-modified polystyrene latex nanoparticles
DHE: Dihydroethidium
GSH: Glutathione
H2-DCFDA: 2′,7′-dichlorodihydrofluorescein diacetate
LDH: Lactate dehydrogenase assay
MAC: Primary human alveolar macrophages
MTT: 3-(4,5-Dimethylthiazol-2-yl)-2,5-diphenyltetrazolium bromide
NAC: N-acetyl cysteine
NP: Nanoparticles
PBS: Phosphate buffered saline
ROS: Reactive oxygen species
SEM: Scanning electron microscopy
SD: Standard deviation
TEM: Transmission electron microscopy
UNP: Unmodified polystyrene latex nanoparticles
