# Supplementary material for: Differential bioreactivity of neutral, cationic and anionic polystyrene nanoparticles with cells from the human alveolar compartment: robust response of alveolar type 1 epithelial cells
Source: Part Fibre Toxicol. 2015 Jul 2;12:19. doi: 10.1186/s12989-015-0091-7 (PMC4489088; doi:10.1186/s12989-015-0091-7)
Supplement: Additional file 1: Figure S1. — Viability of TT1, AT2 and MAC following exposure to 50 nm NPs for 4 h. Low toxicity was observed in TT1 and MAC exposed to high concentrations of ANPs at t = 4 h. At the low concentrations of 1–25 μg/ml ANPs did not show significant toxicity in any cell type. *p < 0.05, n = 3 replicates TT1, and 6 subject samples AT2 and MACs. Figure S2. Effect of adding the antioxidant N-acetyl cysteine to the 50 nm nanoparticles on cell viability. TT1 (a-b), AT2 (c-d) and MAC (e-f) were exposed to 50 μg/ml of 50 nm UNP (a, c and e) and CNP (b, d and f). There was no cytotoxicity on exposure to the NPs, neither was there a significant effect of NAC. n = 3 replicates TT1 and 6 subject samples AT2 and MAC. Figure S3. Release of lactate dehydrogenase following exposure of AT2 cells to 50 and 100 nm UNPs, CNPs and ANPs. The data are expressed as percent of the total cellular LDH in the unexposed control cells. The 50 nm NPs caused release of LDH which was significantly increased relative to NP concentration on exposure to CNP and ANP (b-c; **p < 0.001, n = 6 subject samples). Exposure to 100 nm UNP and CNP had no effect on LDH release (d-e), whereas 100 nm ANP induced significant release of LDH in a concentration dependent manner (f). The 50 nm ANP induced the highest level of LDH release compared to all other NPs. C, **p < 0.001, n = 6 subject samples. Figure S4. Release of lactate dehydrogenase following exposure of MACs to 50 and 100 nm UNPs, CNPs and ANPs. There was significant, NP concentration dependent release of LDH by MACs following exposure to all 100 nm NPs (d-f), which was most significant following ANP (f; **p < 0.001, n = 6 subject samples). Although all types of 50 nm NPs caused some release of LDH, this was only significant for ANP (c); the response was NP concentration dependent but did not reach the same level as that following 100 ANP exposure (c, f; **p < 0.001, n = 6 subject samples). Figure S5. Release of IL-6 and IL-8 following exposure of AT2 cells to UNPs, CNPs [file 12989_2015_91_MOESM1_ESM.pptx]

## Slide 1
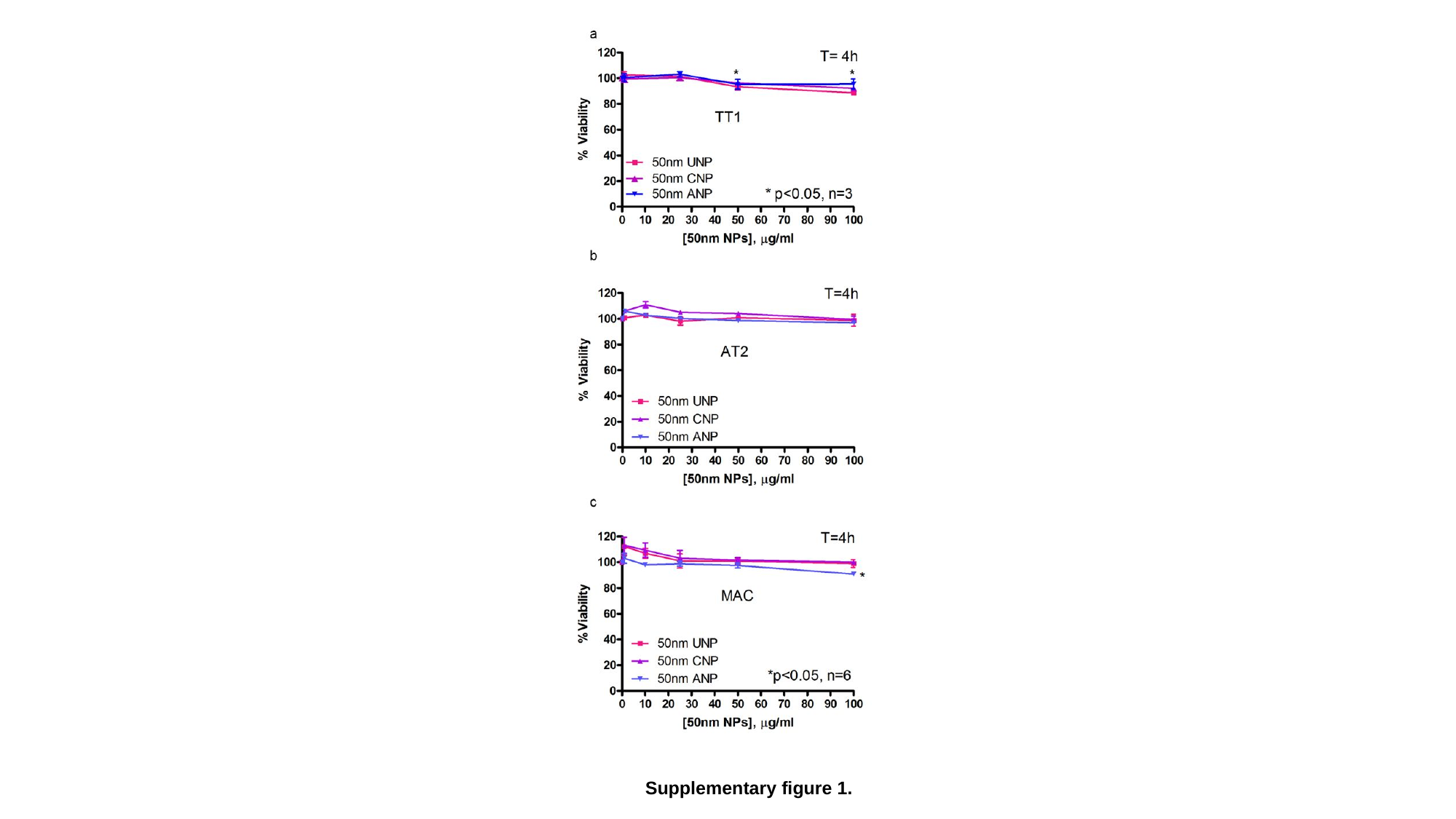

Supplementary figure 1.

## Slide 2
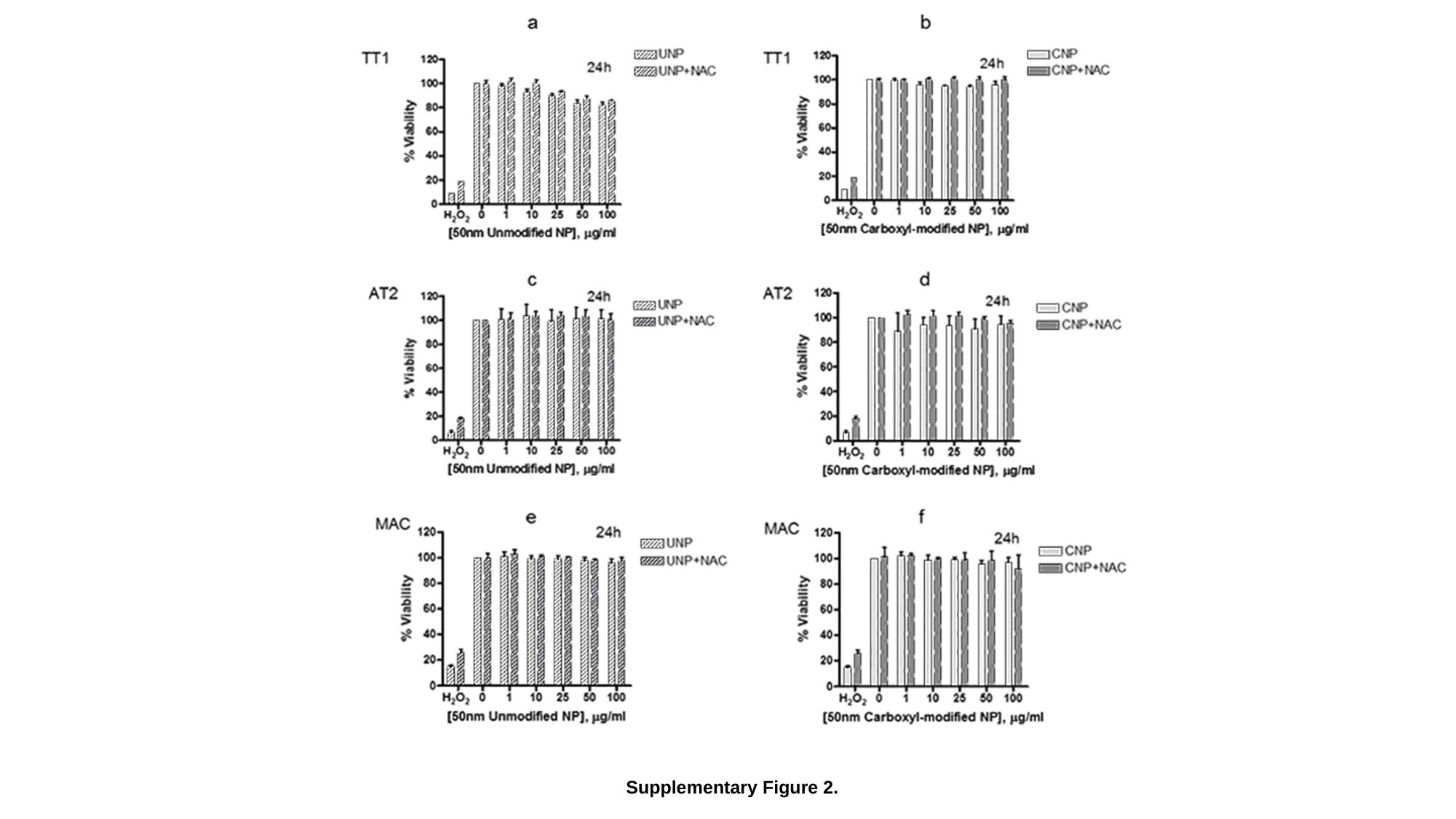

Supplementary Figure 2.

## Slide 3
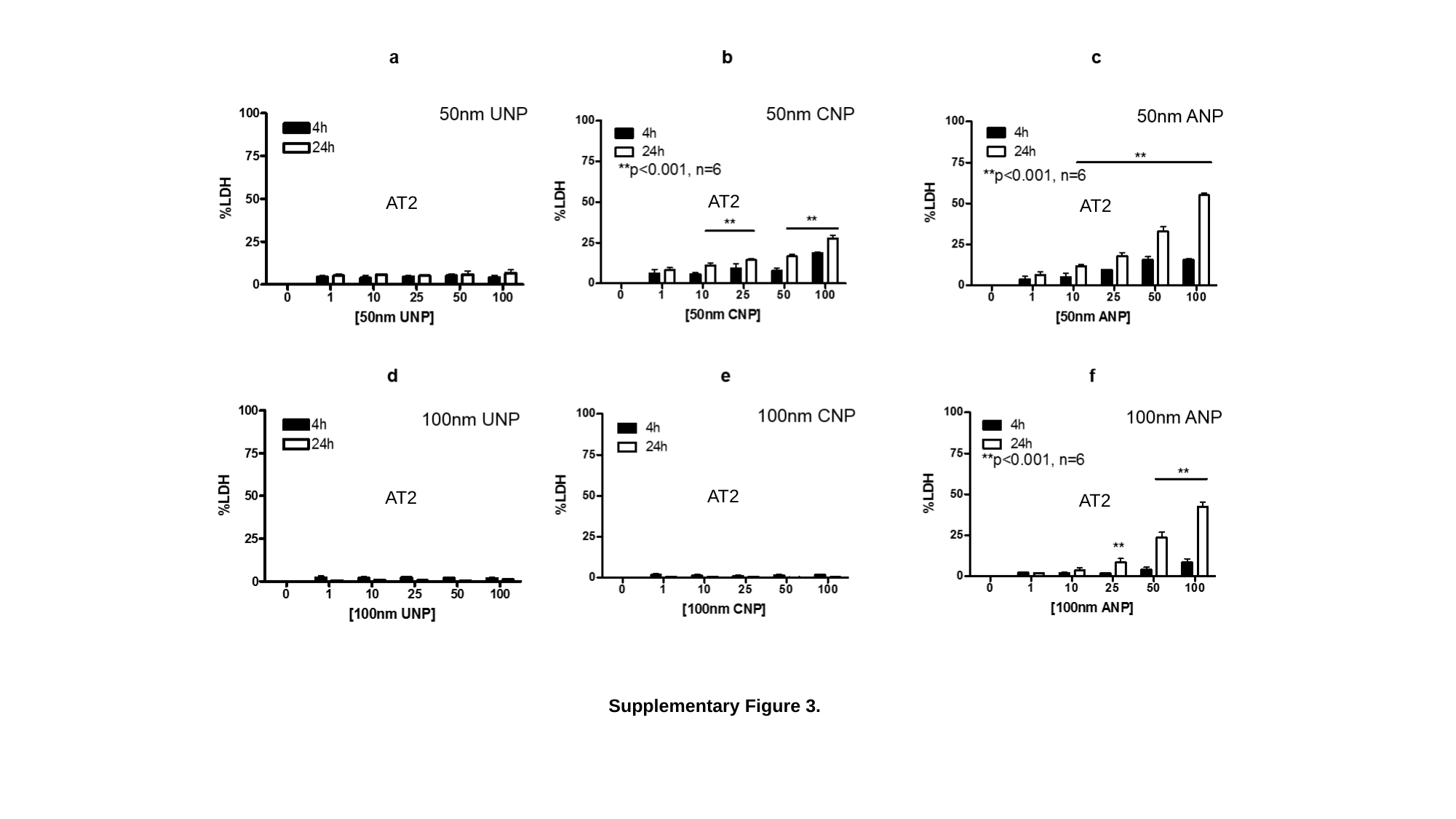

AT2
AT2
AT2
AT2
AT2
AT2
 Supplementary Figure 3.

## Slide 4
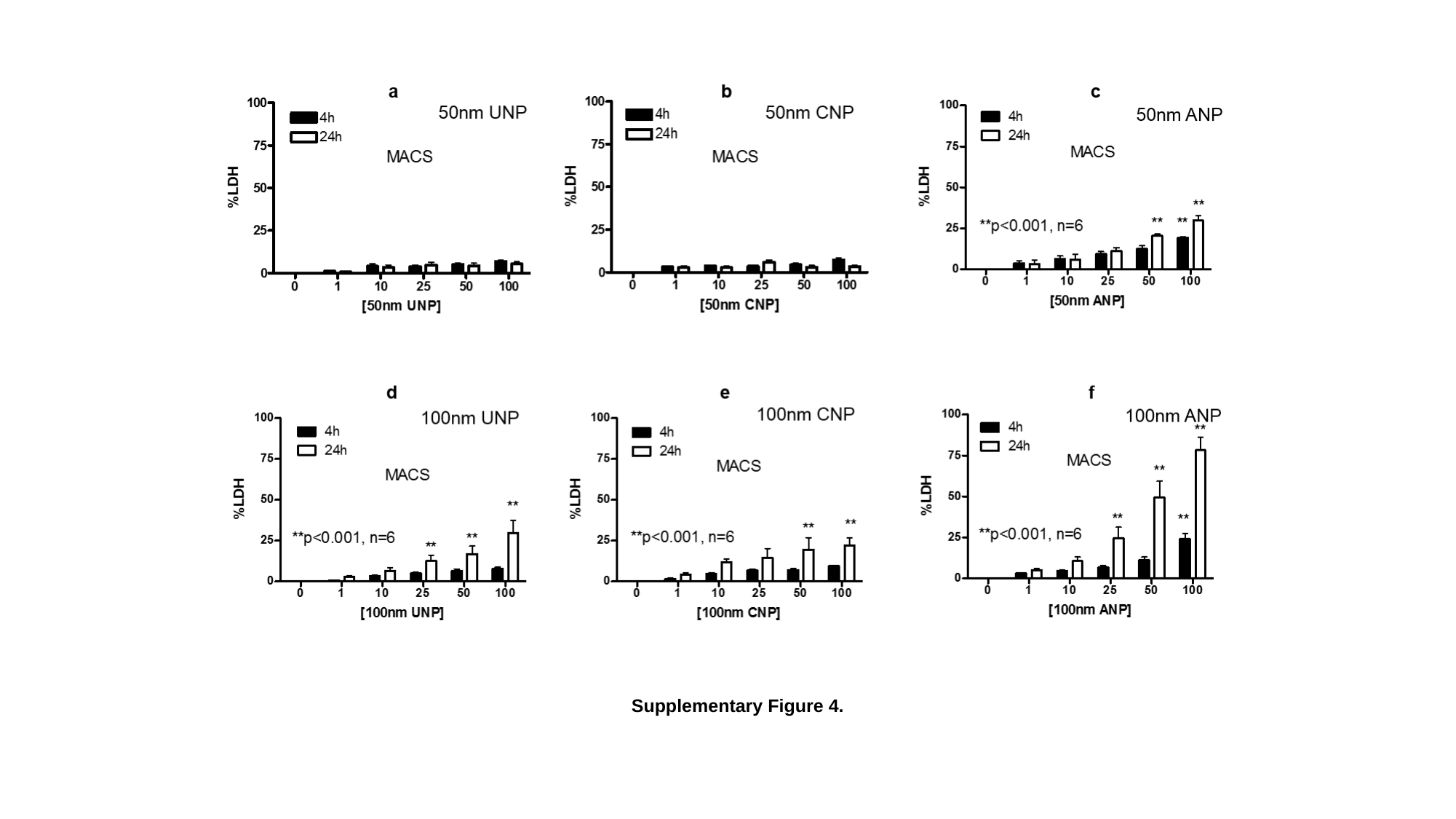

Supplementary Figure 4.

## Slide 5
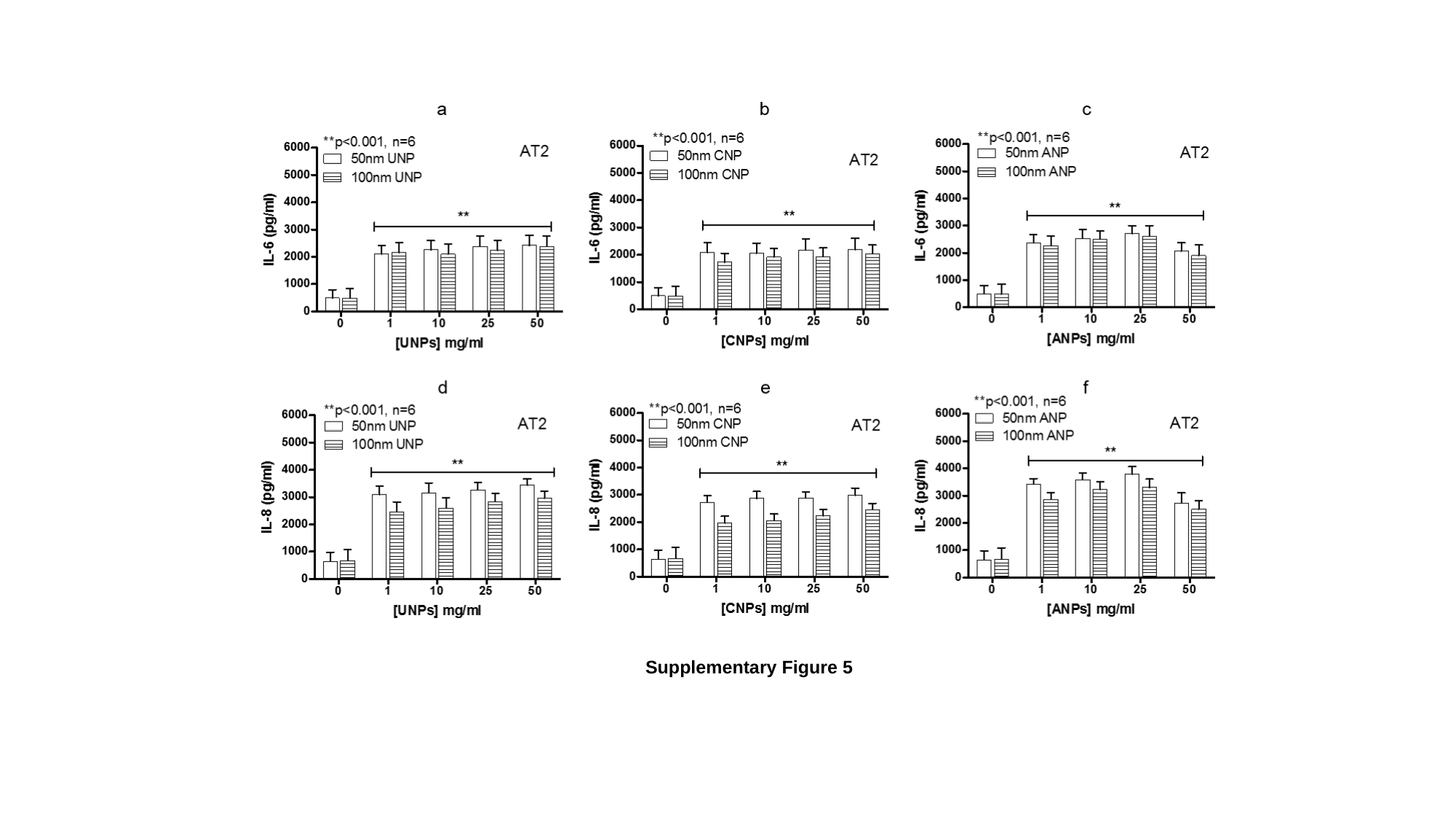

Supplementary Figure 5

## Slide 6
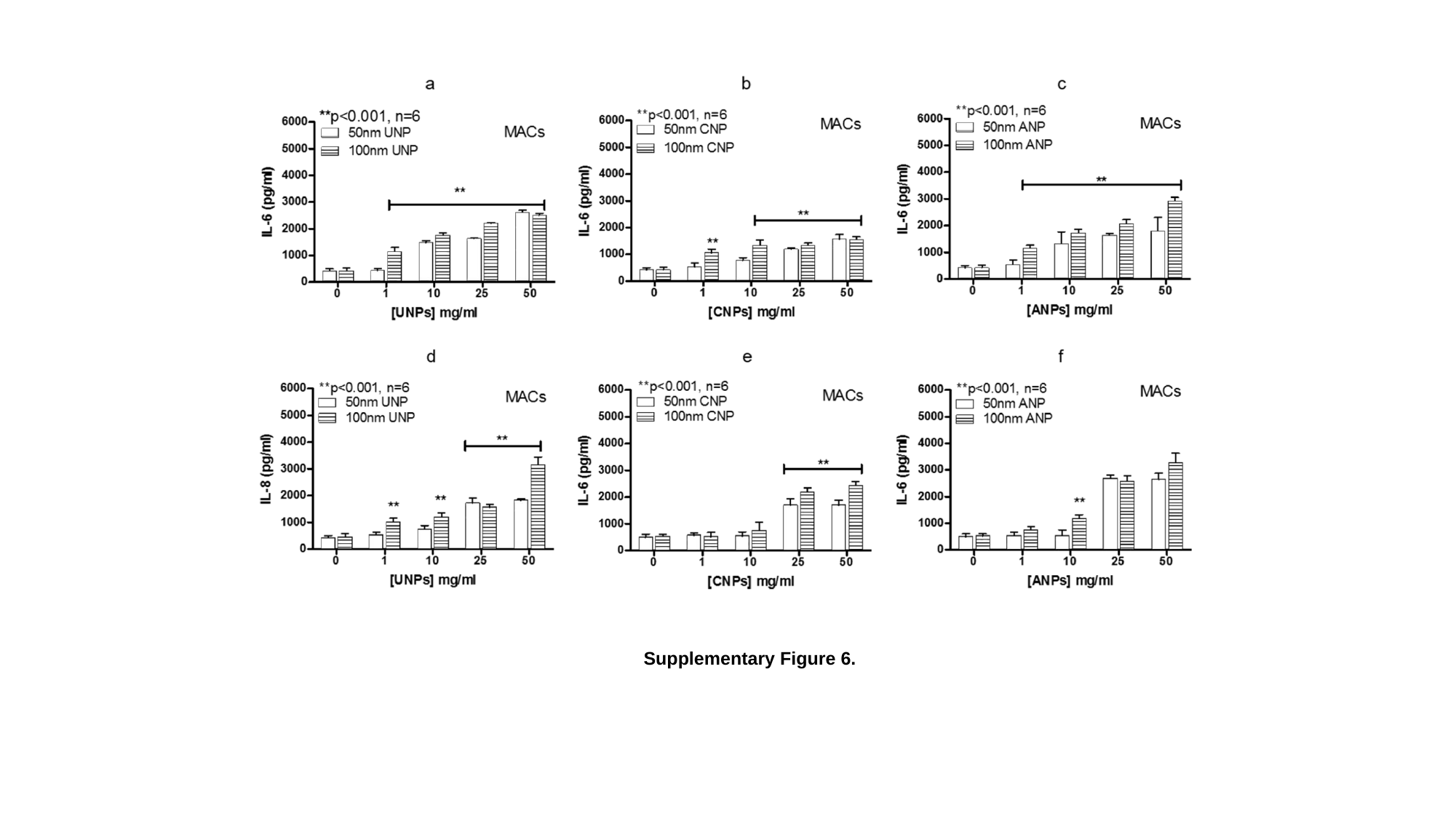

Supplementary Figure 6.

## Slide 7
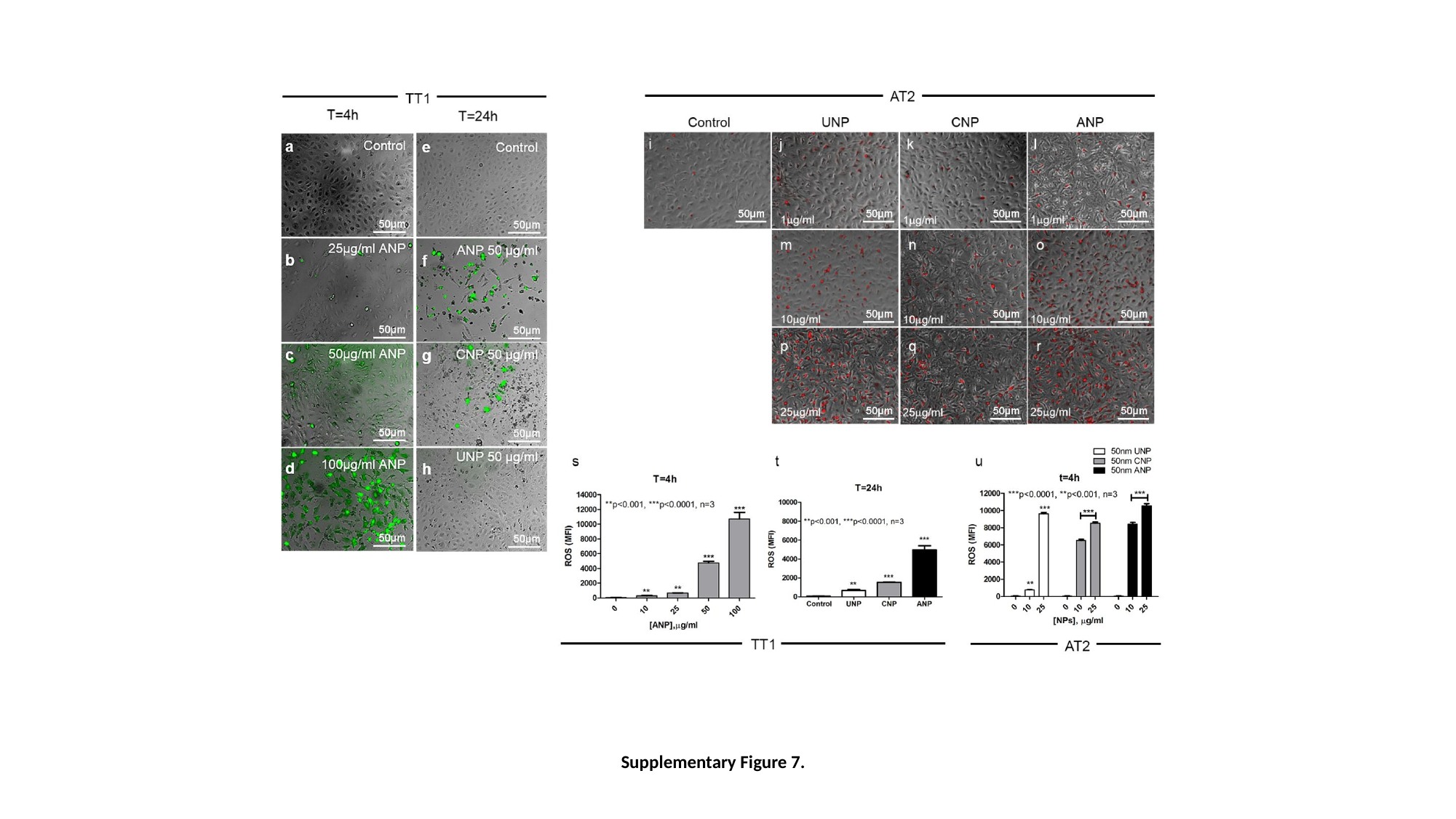

Supplementary Figure 7.
